# Intrachromosomal Amplification, Locus Deletion and Point Mutation in the Aquaglyceroporin AQP1 Gene in Antimony Resistant *Leishmania (Viannia) guyanensis*

**DOI:** 10.1371/journal.pntd.0003476

**Published:** 2015-02-13

**Authors:** Rubens Monte-Neto, Marie-Claude N. Laffitte, Philippe Leprohon, Priscila Reis, Frédéric Frézard, Marc Ouellette

**Affiliations:** 1 Centre de Recherche en Infectiologie du Centre de Recherche du CHU Québec and Département de Microbiologie, Infectiologie et Immunologie, Faculté de Médecine, Université Laval, Québec, Québec, Canada; 2 Departamento de Fisiologia e Biofísica, Instituto de Ciências Biológicas, Universidade Federal de Minas Gerais, Belo Horizonte, Minas Gerais, Brasil; University of Tokyo, JAPAN

## Abstract

**Background:**

Antimony resistance complicates the treatment of infections caused by the parasite *Leishmania*.

**Methodology/Principal Findings:**

Using next generation sequencing, we sequenced the genome of four independent *Leishmania guyanensis* antimony-resistant (SbR) mutants and found different chromosomal alterations including aneuploidy, intrachromosomal gene amplification and gene deletion. A segment covering 30 genes on chromosome 19 was amplified intrachromosomally in three of the four mutants. The gene coding for the multidrug resistance associated protein A involved in antimony resistance was also amplified in the four mutants, most likely through chromosomal translocation. All mutants also displayed a reduced accumulation of antimony mainly due to genomic alterations at the level of the subtelomeric region of chromosome 31 harboring the gene coding for the aquaglyceroporin 1 (LgAQP1). Resistance involved the loss of *LgAQP1* through subtelomeric deletions in three mutants. Interestingly, the fourth mutant harbored a single G133D point mutation in LgAQP1 whose role in resistance was functionality confirmed through drug sensitivity and antimony accumulation assays. In contrast to the *Leishmania* subspecies that resort to extrachromosomal amplification, the *Viannia* strains studied here used intrachromosomal amplification and locus deletion.

**Conclusions/Significance:**

This is the first report of a naturally occurred point mutation in AQP1 in antimony resistant parasites.

## Author Summary

Drug resistance remains a major concern in leishmaniasis chemotherapy, a neglected tropical disease that causes 60,000 deaths around the world annually. To better understand the molecular mechanisms behind drug resistance, we selected *L*. *guyanensis* parasites resistant to antimony, the first-line drug against this disease in many countries. Through whole-genome sequencing we found variations in the copy number of chromosomes in addition to gene amplification and gene deletion events in antimony-resistant parasites. A marker previously related to antimony resistance, the gene coding for multidrug resistant protein A, was found to be amplified. Transport studies revealed a reduced antimony accumulation in resistant parasites that we correlated with the deletion of the gene coding for the aquaglyceroporin 1 (AQP1) responsible for antimony uptake in *Leishmania*. Additionally, a point mutation in *AQP1* was found to be associated with antimony resistance. These findings may contribute to the development of new chemotherapy strategies against leishmaniasis.

## Introduction

Leishmaniasis defines a spectrum of infectious diseases caused by protozoan parasites belonging to the genus *Leishmania* that are transmitted to mammals via the bite of sandflies. Leishmaniasis are neglected tropical diseases that could potentially affect ~ 350 million people in 98 countries [1]. Clinical manifestations differ widely depending on the host immune response and the *Leishmania* species responsible for infection and vary from visceral leishmaniasis—VL to cutaneous leishmaniasis—CL [2]. The clinical manifestations of CL can further vary from localized ulcerative skin lesions to destructive mucosal inflammation (mucocutaneous leishmaniasis—MCL), the latter being mostly associated with infections caused by the *Viannia* subgenus in South America [3–6].

No vaccine is available against leishmaniasis and chemotherapy thus represents the main strategy for the treatment of all forms of the disease [7]. Despite the introduction of paromomycin [8], amphotericin B [9] and miltefosine [10] in the anti-*Leishmania* arsenal, pentavalent antimony (Sb^V^)-derived compounds have been used for more than 65 years and are still the first-line of treatment against leishmaniasis in many countries [11]. Drug combinations, short therapeutic schemes and single drug doses are solutions currently debated to avoid drug resistance, one of the major drawback against leishmaniasis especially in the case of antimony [12]. Antimonial resistance has first emerged against VL in India [13] but cases of treatment failure involving species from the *Viannia* subgenus have since been reported in Brazil [14,15], Peru [16] and Colombia [17]. Drug susceptibility screenings also supported the notion that antimony resistant *L*. (*Viannia*) parasites can develop in the field [18,19].

Antimony is most active against *Leishmania* in its trivalent form (Sb^III^) which is produced through the reduction of pentavalent antimony (Sb^V^) possibly within the macrophage hosts [20] but also within *Leishmania* [21,22]. Sb^III^ is then passively transported into *Leishmania* cells through aquaglyceroporin 1 (AQP1), a porin also allowing the transport of water, glycerol, urea, dihydroxyacetone, methylglyoxal and polyols [23]. Sb^III^ is indeed a chemical mimic of natural AQP1 substrates, having a similar conformation and charge as glycerol [24]. AQP1 plays an important role in volume regulation and osmotaxis in *Leishmania* [25] and its reduced expression is associated with Sb^III^ resistance [26]. On the other hand, re-sensitization is achieved when AQP1 is overexpressed in resistant parasites deficient for AQP1 [26,27]. Targeted mutagenesis of *L*. *major* AQP1 demonstrated a role for residues Glu125 and Ala163 located at the extracellular loop in Sb^III^ susceptibility [28].

While several molecular mechanisms leading to antimony resistance in *Leishmania* have been described, resistance remains only partly understood and most likely constitutes a multifactorial process [29]. Next generation sequencing has been used to produce several *L*. *donovani* genomes and revealed genomic alterations and plasticity that correlated with antimony resistance [30,31]. Gene amplification is also frequently observed in both laboratory-raised or field isolates resistant to antimony, in which circular or linear extrachromosomal DNA are formed by homologous recombination and annealing of direct or inverted repeated sequences, respectively [32]. A well-studied example of such amplification is the gene coding for the multidrug resistance associated protein A (*MRPA*) which is frequently amplified as part of circular amplicons originating from chromosome 23 in Sb^III^-resistant strains [33–35] and whose role in resistance involves the intravesicular sequestration of Sb-thiol conjugates in Sb^III^-resistant *Leishmania* [36].

Our understanding of drug resistance mechanisms come from the analysis of parasites belonging to *Leishmania* subgenus and little is known about the mechanisms leading to antimony resistance in the *Viannia* group, with the exception of few recent studies that highlighted previously observed alterations [37,38]. In this study, whole-genome sequencing was performed in laboratory-selected antimony resistant (SbR) *Leishmania* (*Viannia*) *guyanensis* mutants aiming at the dissection of molecular mechanisms of Sb^III^ resistance in *Leishmania (Viannia)* parasites.

## Methods

### *Leishmania* cultures and in vitro selection of Sb^III^ resistance

*Leishmania* (*Viannia*) *guyanensis* (MHOM/BR/1975/M4147) promastigotes were axenically maintained in minimum essential culture medium (α-MEM) (Gibco, Invitrogen, Grand Island, NY, USA) at pH 7.0 supplemented with 10% (v/v) heat inactivated fetal bovine serum (Wisent Inc., St-Jean-Baptiste, QC, CA), 100 mg mL^-1^ kanamycin, 50 mg mL^-1^ ampicillin, 2 mM L-glutamine, 5 mg mL^-1^ hemin, 5 mM biopterin, (Sigma-Aldrich, St Louis, MO, USA) and incubated at 25°C. Four *L*. *guyanensis* Sb^III^-resistant mutants (LgSb^III^650.1 to LgSb^III^650.4) were independently selected from WT *L*. *guyanensis* in 25 cm^2^ flasks containing 5 mL of α-MEM in the presence of increasing Sb^III^ concentrations. Potassium antimonyl tartrate (Sigma-Aldrich, St Louis, MO, USA) was used as the source of Sb^III^. The stepwise drug selection ranged from 80 μM up to 650 μM of Sb^III^. Last-level SbR mutants were grown in absence of drug pressure for 26 passages to revert resistance. In addition, two independent *L*. *guyanensis* Sb^III^-resistant mutants (LgSb^III^.1/2013 and LgSb^III^.2/2013) were selected by Sb^III^ increments (resistant to 80, 160, 240, 325 or 650 μM Sb^III^) and maintained in culture. For drug susceptibility assay, 10^6^ parasites mL^-1^ in mid-log phase growth were seeded in 24-wells cell culture plates containing 1.5 mL of α-MEM, incubated under gentle agitation at 25°C during 72 h in presence or absence of several concentrations of drug. Growth was monitored daily by measuring absorbance at 600 nm to obtain the Sb sensitivity profile [39].

### Next-generation sequencing

Sequencing libraries were produced from 50 ng of phenol-extracted/ethanol-precipitated genomic DNA by using the Nextera DNA sample preparation kit (Illumina Inc, San Diego, CA, USA) according to manufacturer instructions. Genome sequences were determined by Illumina HiSeq1000 101-nucleotides paired-end reads. Sequencing reads were aligned to a *Leishmania* (*Viannia*) *braziliensis* (MHOM/BR/1975/M2904) reference genome (TriTrypDB version 6.0) [40] using the software package Burrows-Wheeler Alignment [41]. The maximum number of mismatches was 4, the seed length was 32 and 2 mismatches were allowed within the seed. The detection of single nucleotide polymorphisms was performed using SAMtools (version 0.1.18), bcftools (distributed with SAMtools) and vcfutils.pl (distributed with SAMtools) [42]. Putative SNPs detected by whole genome sequencing were verified by conventional PCR amplification and DNA sequencing. Sequencing data are available at the EMBL-EBI European Nucleotide Archive (http://www.ebi.ac.uk/ena) under study accession number PRJEB6114 with samples ERS434587, ERS434588, ERS434589, ERS434590 and ERS434591 corresponding to *L*. *guyanensis* WT, LgSb^III^650.1, LgSb^III^650.2, LgSb^III^650.3 and LgSb^III^650.4, respectively.

### Real time RT-PCR

First-strand cDNA was synthesized from 5 μg of total RNA using Oligo dT12–18 and SuperScript II RNase H-Reverse Transcriptase (Invitrogen, Carlsbad, CA, USA) according to the manufacturer protocol. Equal amounts of cDNA were run in triplicate and amplified in 25 μL reactions containing 1 x iQ SYBR Green Supermix (Bio-Rad, Hercules, CA, USA), 100 nM forward and reverse primers and 100 ng of cDNA target. Reactions were carried out using a rotator thermocycler Rotor Gene (RG 3000, Corbett Research, San Francisco, USA). Mixtures were initially incubated at 95°C for 5 min and then cycled 30 times at 95°C, 60°C and 72°C for 15 s. No-template controls were used as recommended. Three technical and biological replicates were established for each reaction. The relative amount of PCR products generated from each primer set was determined based on the cycle threshold—Ct value and the amplification efficiencies. Data were analyzed using the comparative 2^-ΔΔCt^ method. Gene expression levels were normalized to constitutively expressed mRNA encoding glyceraldehyde-3-phosphate dehydrogenase (*GAPDH*, *LbrM*.*30*.*2950*). Primers for targeted genes and internal gene expression control *GAPDH* were designed using PrimerQuest (http://www.idtdna.com/Primerquest/Home/Index) and sequences are listed in S1 Table.

### PFGE and Southern blot

Molecular karyotype was obtained from *L*. *guyanensis* WT and Sb-resistant mutants by separation of chromosomes by pulse field electrophoresis [43]. 10^8^ mid-log phase parasites were embedded in low melting point agarose blocks, digested with proteinase K and electrophoresed in a contour clamped homogenous electric field apparatus (CHEF Mapper, Bio-Rad, Hercules, CA, USA). The blocks were mounted in 1% agarose gel and electrophoresed in 0.5x Tris-Borate-EDTA running buffer at 5 V cm^-1^ with 120° separation angle at 14°C during 30 h. A range of 150 to 1500 kb was applied for a wide chromosomal separation, resolving most of *Leishmania* chromosomes in a single molecular karyotype gel. *Saccharomyces cerevisiae* chromosomes were used as DNA size marker (Bio-Rad, Hercules, CA, USA). For Southern blots, genomic DNA was isolated using DNAzol reagent (Life Technologies, Carlsbad, CA, USA) following manufacturer’s instructions and digested with the *Pst*I restriction enzyme (New England Biolabs Inc, Ipswich, MA, USA). Digested genomic DNA or PFGE-derived molecular karyotype were transferred by capillarity onto nylon membranes (Hybond-N+, Amersham Pharmacia Biotehc, Sunnyvale, CA, USA) and cross-linked with UV light. The blots were hybridized with [α-^32^P]dCTP labeled DNA probes according to standard protocols [44]. Primers used for southern blot probes are listed in S1 Table. Densitometric quantification of southern blot-derived bands was performed using Image J version 1.48a.

### Cloning and transfection of *LgAQP1* in *Leishmania*

The gene LgAQP1 (GenBank accession numbers KJ623262 and KJ623263) was amplified from genomic DNA of *L*. *guyanensis* WT and LgSb^III^650.4 using primers containing 5’ *Xba*I and 3’ *Hind*III restriction sites, followed by cloning in pGEM T-easy (Promega, Madison, WI, USA). The WT *AQP1* and its LgSb^III^650.4 variant were subcloned into the pSP72αZEOα expression vector, a derivative of pSP72αNEOα [45] in which the gene neomycin phosphotransferase (NEO) was replaced by the bleomycin-binding protein gene (ZEO) conferring resistance to zeocin [46]. To validate the expression of episomal LgAQP1, a green fluorescent protein (GFP)-tagged construct was made using a PCR fusion-based strategy as previously described [47] using primers listed in S1 Table. The GFP gene was amplified using the pSP72αNEOαGFP vector as template. The LgAQP1-GFP fusions were cloned into pGEM T-easy and subcloned into the *Xba*I/*Hind*III sites of pSP72αZEOα, resulting in the pSP72αZEOαLgAQP1WTGFP or pSP72αZEOαLgAQP1(G133D)GFP constructs that were transfected by electroporation as previously reported [45]. Transfected parasites were preselected in the presence of 500 μg mL^-1^ of Zeocin Selection Reagent (Life Technologies, Carlsbad, CA, USA) and after 24 h, selection of transfectants was carried out in presence of 1 mg mL^-1^ of Zeocin Selection Reagent. Before transfection, all constructs were confirmed by DNA sequencing.

### Western blot

Total and membrane protein fractions were extracted from *Leishmania* as previously described [48]. Briefly, parasites were centrifuged and washed three times with ice-cold Hepes-NaCl at 3000 rpm for 5 minutes. The pellet was resuspended in a lysis buffer (10 mM Tris-HCl pH 7.4, 10 mM NaCl, 1.5 mM MgCl_2_, 1 mM DTT) and homogenized by vortexing after addition of proteases inhibitors cocktail (1 mg mL^-1^ leupeptin, 2 μg mL^-1^ aprotinine, 5 mM EDTA). The lysate was then incubated on ice for 15 min, followed by three cycles of freeze (-80°C) and thaw (37°C). The supernatant was recovered after centrifugation at 15000 rpm for 30 min at 4°C. From there, supernatant containing membrane fractions was kept at -80°C. Proteins were then extracted from membranes fractions using solubilisation buffer (50 mM Tris-HCl pH 8, 150 mM NH_4_Cl, 2 mM MgCl_2_, CHAPS 1%) by incubation on ice for 30 min. 50 μg of proteins were run on 10% acrylamide gel and transferred electrically onto nitrocellulose membrane (Bio-Rad, Hercules, CA, USA). The blots were blocked overnight in PBS (1 X), Tween (0.1%), Milk (5%). Membranes were incubated overnight at 4°C with a GFP monoclonal antibody (Roche, Basel, Switzerland) and an α-tubulin monoclonal antibody (Life Technologies, Carlsbad, CA, USA) diluted 1:1000 in PBS-Tween-Milk solution. Membranes were then washed three times for 5 min in PBS-Tween and incubated 1 h with horseradish peroxidase-conjugated goat anti-mouse IgG (Thermo Fisher Scientific Inc, Waltham, MA, USA) diluted 1:10000 in PBS-Tween. Membranes were washed again three times and incubated with Immobilon western chemiluminescent HRP substrate (Millipore, Billerica, MA, USA).

### Antimony uptake assay

Antimony accumulation measurements were carried out based on previous studies [49,50]. Briefly, 10^8^ mid-log phase *Leishmania* promastigotes were washed and resuspended in 1 mL of Hepes/NaCl/Glucose buffer (20 mM HEPES, 0.15 M NaCl, 10 mM glucose, pH 7.2) followed by incubation with 540 μM Sb^III^ at 25°C as previously described [38]. One hour of Sb incubation was chosen to compare differences in Sb accumulation among the conditions evaluated. Drug accumulation was stopped by incubating cells on ice followed by three washes with ice-cold Hepes/NaCl/Glucose buffer. Parasites were centrifuged at 1800 g during 5 min at 4°C and the dried pellet was digested in 100 μL of 65% HNO_3_ (Merck, Darmstadt, Germany) before Sb was quantified by graphite furnace electrothermal atomic absorption spectrometry using an AAnalyst 600/800 spectrometer (Perkin Elmer, Waltham, MA, USA). Blank matrix was established by measuring Sb traces in Sb-unexposed HN0_3_-digested cells. Blank absorbance values were subtracted as background. Intracellular Sb content was normalized by number of cells. Sb-resistant *Leishmania* mutants were maintained at least 2 passages without drug pressure prior to the transport assay to avoid contaminations.

### Statistical analyses

EC_50_ values were calculated by non-linear regression when applied, data were analyzed by Student’s *t* test or analysis of variance (ANOVA) followed by correction performed using Bonferroni’s multiple comparison test. A *p* value ≤ 0.05 were considered statistically significant. Statistical analyses were carried out using the software GraphPad Prism version 5.0 (GraphPad Software Inc., La Jolla, CA, USA).

## Results

### *In vitro* selection of Sb-resistant *L*. *guyanensis* parasites

Four independent SbR *L*. *guyanensis* mutants (LgSb^III^650.1, LgSb^III^650.2, LgSb^III^650.3 and LgSb^III^650.4) were obtained *in vitro* by stepwise Sb^III^ selection. While *L*. *guyanensis* wild-type (WT) parasites presented an EC_50_ of 53.72 μM, the four SbR mutants were resistant to at least 1 mM of Sb^III^, representing a resistance index (RI) of more than 18 times (Table 1). The resistance phenotype of every mutant remained stable even after 26 passages in absence of antimony, at which point parasites still presented Sb^III^ EC_50_ values superior to 1 mM (Table 1).

**Table 1:** Half-maximal effective concentrations (EC_50_) of Sb^III^ and correspondent resistance indexes (RI) for *L*. *guyanensis*.

Strain	Sb^III^ EC_50_ (μM) ± SEM	RI
*L guyanensis* M4147 WT	53.72 ± 0.12	
LgSb^III^650.1	>1000	>18.6
LgSb^III^650.2	>1000	>18.6
LgSb^III^650.3	>1000	>18.6
LgSb^III^650.4	>1000	>18.6
		
LgSb^III^650.1 rev	>1000	>18.6
LgSb^III^650.2 rev	>1000	>18.6
LgSb^III^650.3 rev	>1000	>18.6
LgSb^III^650.4 rev	>1000	>18.6
		
LgSb^III^80.1/2013	318.4 ± 2.95	6
LgSb^III^80.2/2013	221.8 ± 2.25	4.12
LgSb^III^160.1/2013	450.5 ± 2.87	8.4
LgSb^III^160.2/2013	>1000	>18.6
LgSb^III^240.1/2013	853.4 ± 3.4	15.8
LgSb^III^240.2/2013	966.3 ± 4.6	18
LgSb^III^325.1/2013	>1000	>18.6
LgSb^III^325.2/2013	>1000	>18.6
LgSb^III^650.1/2013	>1000	>18.6
LgSb^III^650.2/2013	>1000	>18.6
		
LgSb^III^650.2 + pSP72αZEOαLgAQP1WTGFP	29.09 ± 0.35	0.54
LgSb^III^650.2 + pSP72αZEOαLgAQP1G133DGFP	>1000	>18.6
LgSb^III^650.2 + pSP72αZEOα	>1000	>18.6
		
LgSb^III^650.2 + pSP72αZEOαLgAQP1WT	31.4 ± 0.57	0.6
		
LgWT + pSP72αZEOαLgAQP1WT	12.93^a^ ± 0.32	0.24
LgWT + pSP72αZEOαLgAQP1G133D	51.98 ± 0.33	0.96
LgSb^III^650.4 + pSP72αZEOαLgAQP1WT	28.63^b^ ± 0.38	0.53

SEM (standard error of the mean); RI (resistance index)

^a^ 76% increased Sb^III^ sensitivity compared to Lg WT

^b^ At least 97% re-sensitization when compared to LgSb^III^650.4

EC_50_ values are the average of at least three independent experiments.

### Increased gene copy number in *L*. *guyanensis* SbR mutants is due to aneuploidy and to intrachromosomal amplification

Whole-genome sequencing was conducted on the four independent *L*. *guyanensis* SbR lines as well as on the isogenic *L*. *guyanensis* M4147 WT line by Illumina next-generation sequencing. For all strains, this produced genome assemblies of 31 Mb with a coverage depth of at least 50 fold. Copy number variations (CNVs) associated with resistance were identified by comparing the coverage of uniquely mapped reads between *L*. *guyanensis* SbR mutants and the WT line as part of small non-overlapping genomic windows (5 kb) along the chromosomes (normalized for the total number of uniquely-mapped reads for each strain) [51]. This enabled the observation of CNVs at the level of entire chromosomes (aneuploidy) and at specific genomic loci (amplification/deletion).

**Fig 1:**
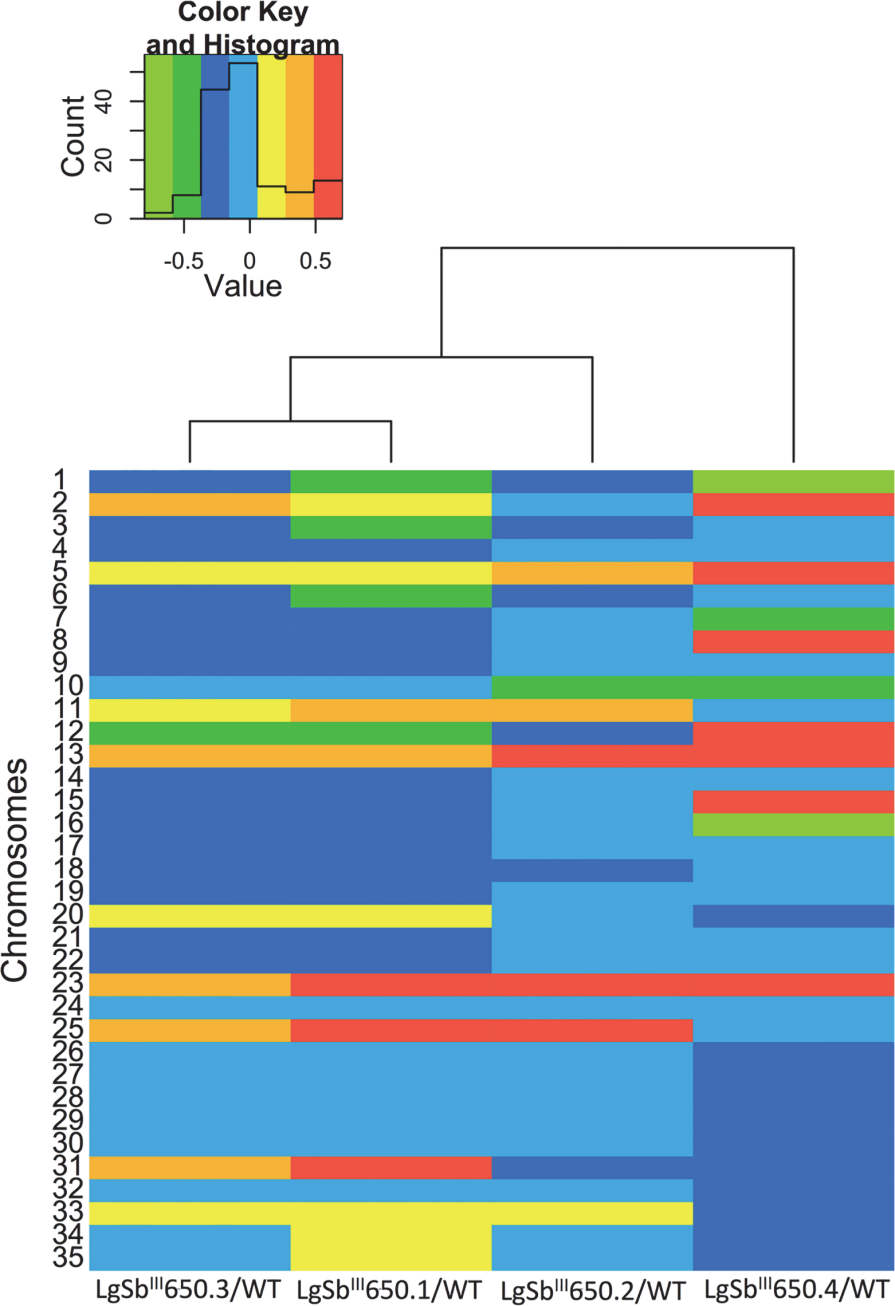
Chromosome copy number variation in antimony resistant *L*. *guyanensis* mutants. Heatmap representation of log_2_-transformed normalized SbR/WT read ratio for all 35 chromosomes in the four independent SbR *L*. *guyanensis* mutants. Chromosomes were divided into non-overlapping 5 kb genomic windows and median SbR/WT reads ratios for each chromosome was normalized according to the total number of reads followed by log_2_-transformation [51]. Intermediate values are represented in the color key inset by a color range varying from green (less copy number) to red (increased copy number). A dendrogram tree groups the mutants according to similarities in CNVs.

Several cases of supernumerary chromosomes were observed in the SbR mutants (Figs. 1 and S1). Most of these had log_2_ SbR/WT read ratios close to 0.5 indicating a gain of about 1.5 chromosome copies compared to WT parasites. Parasites from the *Leishmania Viannia* subgenus are distinct from other *Leishmania* species in harboring predominantly trisomic genomes [52] and this should thus represent a gain of one allele compared to WT parasites (going from 3 to 4 chromosome copies). Most supernumerary chromosomes were not shared by the mutants however; chromosome 13 was consistently increased in all SbR mutants, and chromosomes 11 and 25 were increased in three of the four mutants (LgSb^III^650.1, LgSb^III^650.2 and LgSb^III^650.3) (S1 Fig.). Chromosome losses were also observed in the SbR mutants and these were consistent with the loss of one allele (S1 Fig.). Interestingly, CNVs calculated from read depth coverage often led to a cumulative ploidy not matching with a clear-cut number of chromosomes but instead to intermediate log_2_ SbR/WT values (S1 Fig.). This was observed for both chromosome gains and losses and suggests differences in chromosome-level CNVs between individual cells within the population, a phenomenon known as mosaic aneuploidy [53]. Overall, mutant LgSb^III^650.4 was more divergent and displayed the highest level of chromosome-level CNVs compared to the three other mutants (Fig. 1).

**Fig 2:**
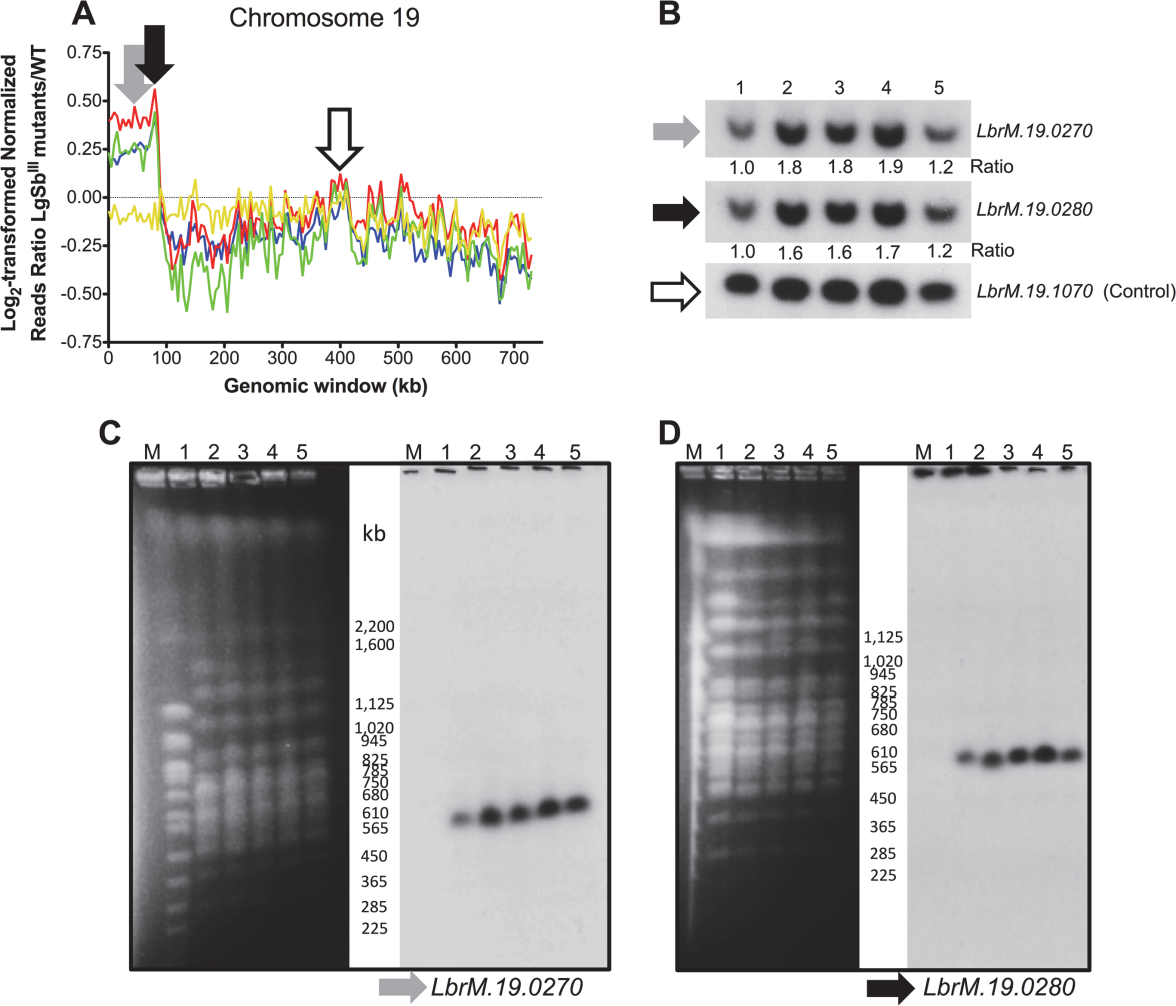
Intrachromosomal amplification in antimony resistant *L*. *guyanensis* mutants. (A) Log_2_-transformed SbR/WT reads ratios for non-overlapping 5 kb genomic windows on chromosome 19. Blue, LgSb^III^650.1; Red, LgSb^III^650.2; Green, LgSb^III^650.3; and Yellow, LgSb^III^650.4. Grey, black and white arrows define the location of probes derived from genes *LbrM*.*19*.*0270*, *LbrM*.*19*.*0270* and *LbrM*.*19*.*1070*, respectively that were used for hybridization of Southern blots in panels B-D. (B) Southern blots of *Pst*I-digested genomic DNA hybridized with probes derived from genes *LbrM*.*19*.*0270*, *LbrM*.*19*.*0280* and *LbrM*.*19*.*1070* (see S1 Table for probe details). Chromosomes were separated by pulsed-field gel electrophoresis and hybridized with (C) *LbrM*.*19*.*0270* and (D) *LbrM*.*19*.*0280* probes. Lanes: M, molecular weight marker; 1, LgM4147 wild type; 2, LgSb^III^650.1; 3, LgSb^III^650.2, 4, LgSb^III^650.3; 5, LgSb^III^650.4.

Normalized read depth coverage allowed the identification of amplified and deleted genomic loci in the *L*. *guyanensis* SbR mutants. These are characterized by punctuated series of genomic windows one beside the other whose normalized read coverage varies compared to the WT baseline [51], as observed for chromosomes 19 and 23 in more than one mutant (S1 Fig.). For chromosome 19, a subtelomeric region of 87.5 kb covering 30 genes (*LbrM*.*19*.*0010* to *LbrM*.*19*.*0300*) appeared to be amplified in mutants LgSb^III^650.1, LgSb^III^650.2, and LgSb^III^650.3 based on normalized read counts (Fig. 2A) and this amplification was confirmed by the hybridization of Southern blots with three distinct probes along the chromosome (Fig. 2A and 2B). Probes derived from genes *LbrM*.*19*.*0270* and *LbrM*.*19*.*0280* (all gene IDs reported in this work are based on the closest *L*. *braziliensis* genome used for alignments of *L*. *guyanensis* sequencing reads) located within the amplified region of chromosome 19 yielded 1.6 to 1.9 fold-increase hybridization intensities for mutants LgSb^III^650.1, LgSb^III^650.2, and LgSb^III^650.3 compared to WT cells after normalization with *LbrM*.*19*.*1070* used as an internal control for DNA loading (Fig. 2B). Consistent with the NGS data, mutant LgSb^III^650.4 had band intensities equivalent to WT for both *LbrM*.*19*.*0270* and *LbrM*.*19*.*0280* (Fig. 2B). Interestingly, for mutants LgSb^III^650.1, LgSb^III^650.2, and LgSb^III^650.3 harboring the subtelomeric amplification on chromosome 19, hybridization of chromosomes separated by PFGE with probes derived from genes *LbrM*.*19*.*0270* and *LbrM*.*19*.*0280* revealed a unique band (corresponding to chromosome 19) supporting intrachromosomal duplication of a specific region rather than extrachromosomal elements.

**Fig 3:**
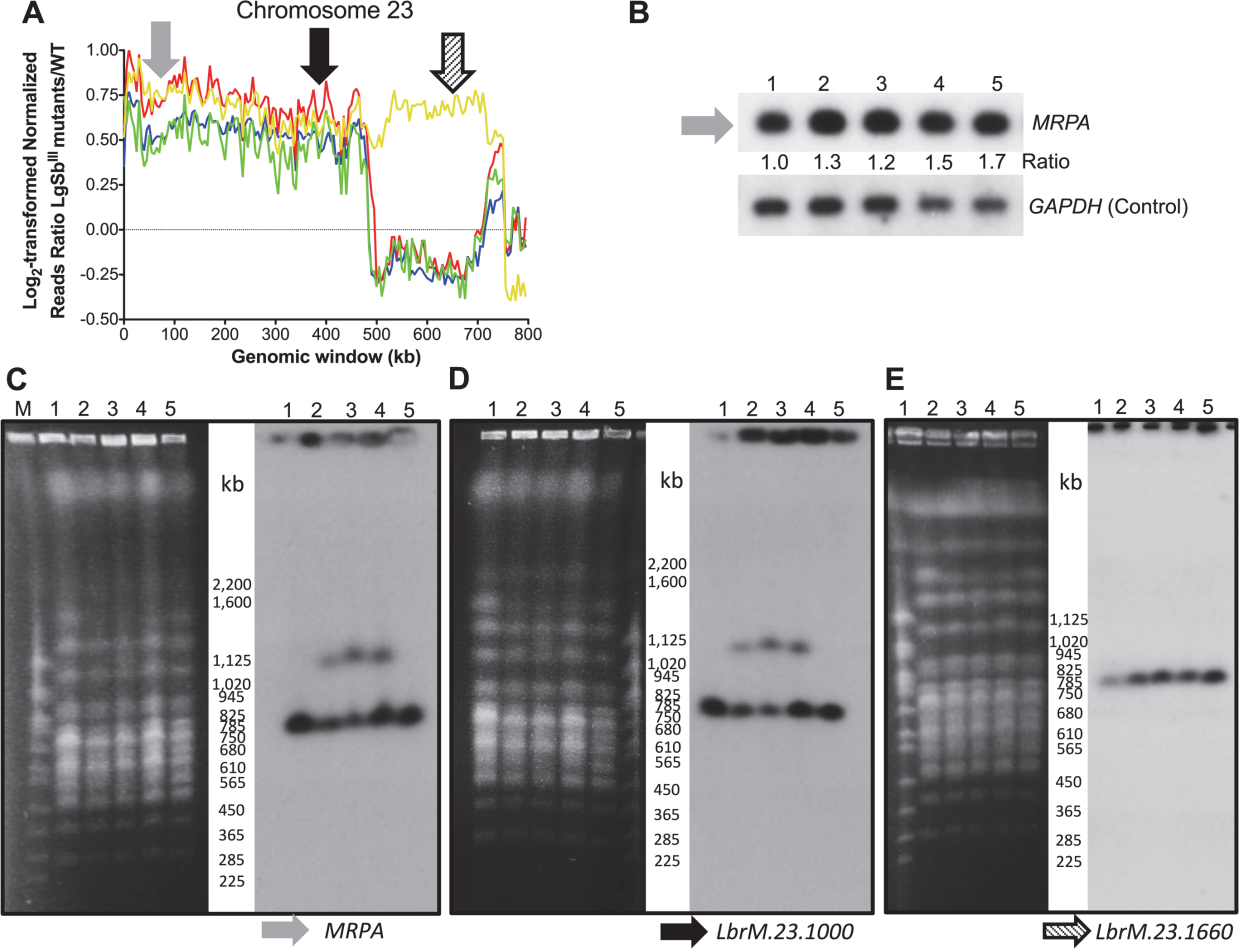
Chromosome 23 amplification in antimony resistant *L*. *guyanensis* mutants. (A) Log_2_-transformed SbR/WT reads ratios for non-overlapping 5 kb genomic windows on chromosome 23. Blue, LgSb^III^650.1; Red, LgSb^III^650.2; Green, LgSb^III^650.3; and Yellow, LgSb^III^650.4. Grey, black and hatched arrows define the location of probes derived from genes *MRPA*, *LbrM*.*23*.*1000*, *LbrM*.*23*.*1660* and *LbrM*.*23*.*1910*, respectively, that were used for hybridization of Southern blots in panels B-E. (B) Southern blot hybridization of *Pst*I-digested genomic DNA with a probe derived from *LbrM*.*23*.*0280* (*MRPA*). The blot was also probed with *GAPDH* and used as a loading control. Southern blots of PFGE-separated chromosomes were hybridized with probes derived from genes *MRPA* (C), *LbrM*.*23*.*1000* (D) and *LbrM*.*23*.*1660* (E). Lanes: M, molecular weight marker; 1, LgM4147 WT; 2, LgSb^III^650.1; 3, LgSb^III^650.2, 4, LgSb^III^650.3; 5, LgSb^III^650.4.

For chromosome 23, the amplified region was much larger than for chromosome 19 and covered 480–495 kb in mutants LgSb^III^650.1, LgSb^III^650.2, and LgSb^III^650.3 (Fig. 3A), starting from one subtelomeric end and encompassing the locus coding for the well-established Sb resistance gene *MRPA* (*LbrM*.*23*.*0280*) (Fig. 3A and 3B). In mutant LgSb^III^650.4, the increased in read length covers (almost) the entire length of the chromosome (Fig. 3A), suggesting an increased in ploidy. Southern blots hybridization of *Pst*1-digested genomic DNA revealed an up to 1.7 fold increased intensity for a *MRPA*-derived probe in the LgSb^III^650.1–4 mutants after normalization with *GAPDH* signals used as DNA loading control (Fig. 3B). This is consistent with NGS data that revealed a 1.4–1.7 increased reads counts in the mutants compared to WT parasites (Fig. 3A). Intriguingly, PFGE-derived Southern blots hybridized with *MRPA* and *Lbr*.*23*.*1000*, two genes comprised in the 480–495 kb region amplified in mutants LgSb^III^650.1 to LgSb^III^650.3, presented a signal at 785 kb corresponding to chromosome 23 but also an additional signal at around 1.1 Mb (Fig. 3C and 3D). This 1.1 Mb band did not hybridize with *LbrM*.*23*.*1660*, a probe outside of the 480–495 kb amplified region (Fig. 3E). It is unclear how a region of chromosome 23 found its way to this chromosome. It is unlikely that it presents a linear amplicon as we never observed such large extrachromosomal elements [54,55] and the hybridization intensity (Fig. 3C and 3D) would suggest that this region has translocated into only one of the two homologous recipient chromosome in mutants LgSb^III^650.1, LgSb^III^650.2 and LgSb^III^650.3 (see also S2 Fig.).

**Fig 4:**
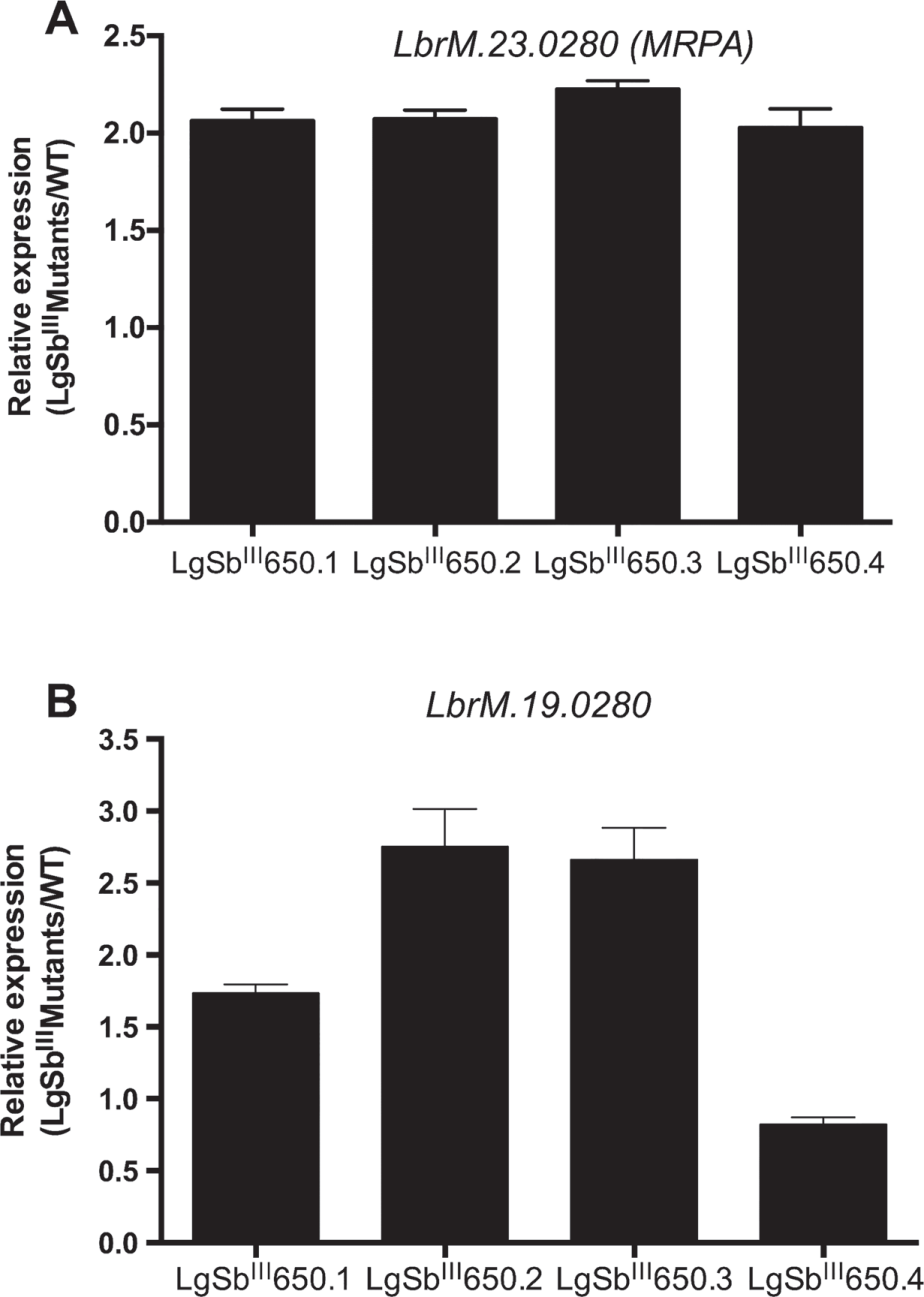
Gene expression correlates with gene copy number in antimony resistant *L*. *guyanensis* mutants. The expression of *MRPA* (A) and *LbrM*.*19*.*0280* (B) in LgSb^III^650.1–4 was compared to WT parasites. The SbR/WT expression ratios were normalized according to *GAPDH* (*LbrM*.*30*.*2950*) levels. Values represent the mean of at least two independent measurements performed with three biological replicates.

Quantitative real time PCR validated that DNA amplification on chromosome 19 and 23 translated into increased mRNA levels (Fig. 4). The four independent mutants presented twice-more mRNA levels for *MRPA* compared to WT (Fig. 4A) while genes on chromosome 19 were upregulated in mutants LgSb^III^650.1, LgSb^III^650.2 and LgSb^III^650.3, but not in LgSb^III^650.4 (Fig. 4B), confirming what was previously observed at the genomic level.

### AQP1-containing locus on chromosome 31 is deleted in three *L*. *guyanensis* SbR mutants

**Fig 5:**
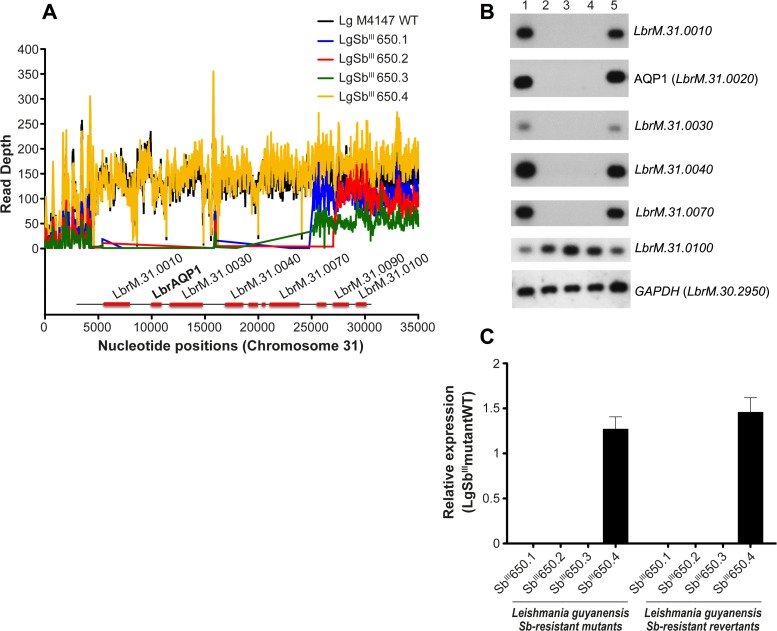
Subtelomeric deletion of chromosome 31 and *LgAQP1* expression in antimony resistant *L*. *guyanensis*. (A) Zoomed representation of raw read depth for one of the subtelomeric region of chromosome 31. The inset scheme indicates the gene positions on the chromosome. Black, LgM4147 WT; Blue, LgSb^III^650.1; Red, LgSb^III^650.2; Green, LgSb^III^650.3; and Yellow, LgSb^III^650.4. (B) Southern blot hybridization validating the subtelomeric deletions of chromosome 31. *PstI*-digested genomic DNAs were hybridized with probes derived from genes located within (*LbrM*.*31*.*0010*—*LbrM*.*31*.*0070*) or outside (*LbrM*.*31*.*0100*) the deleted region. *GAPDH* was used as a qualitative DNA loading control for one of the blots and should not be used for determining changes in gene copy numbers. Lanes: 1, LgM4147 WT; 2, LgSb^III^650.1; 3, LgSb^III^650.2, 4, LgSb^III^650.3; 5, LgSb^III^650.4. (C) Relative *AQP1* mRNA levels in LgSb^III^650.1, LgSb^III^650.2, LgSb^III^650.3 and LgSb^III^650.4 and their revertants compared to WT. Revertants were cultured for at least 26 passages in the absence of Sb^III^. The SbR/WT expression ratios were normalized according to *GAPDH* (*LbrM*.*30*.*2950*) levels. Values are the mean of at least three independent experiments each performed with three biological replicates.

A fine scale analysis of sequencing coverage revealed that a subtelomeric deletion occurred on chromosome 31 in three of the four SbR *L*. *guyanensis* mutants (S1 Fig. and Fig. 5A). The deleted region covered around 25 kb in mutants LgSb^III^650.1 and LgSb^III^650.3 and 27 kb in mutant LgSb^III^650.2 (Fig. 5A). In all three mutants the deleted region harbored the gene coding for the aquaglyceroporin AQP1 (*LbrM*.*31*.*0020*) known to be associated with antimony uptake in *Leishmania* [27]. Interestingly, sequencing reads could still be detected within 5 kb of the end of chromosome 31 in the mutants that presented *AQP1* deletion (Fig. 5A) which could suggest telomere seeding in response to the loss of a terminal part of chromosome 31 (Figs. 5A and S1). The subtelomeric deletion in LgSb^III^650.1, LgSb^III^650.2 and LgSb^III^650.3 was confirmed by hybridization of Southern blots using probes located within and outside the deleted region. As expected, no signal was detected from gene *LbrM*.*31*.*0010* to *LbrM*.*31*.*0070* in mutants LgSb^III^650.1, LgSb^III^650.2 and LgSb^III^650.3 while LgSb^III^650.4 and WT parasites presented a clear *AQP1* signal (Fig. 5B). Conversely, hybridization signals were detected for every strain when the blots were probed with gene *LbrM*.*31*.*0100* located outside the deleted regions or with the *GAPDH* gene located on a distinct chromosome (Fig. 5B). Since *AQP1* was not deleted in mutant LgSb^III^650.4, qRT-PCR assays were carried out in order to infer about any possible regulation of *AQP1* expression in this mutant. However, *AQP1* mRNA levels were similar in WT and in the LgSb^III^650.4 mutant growing in presence of Sb^III^ or in its absence for 26 passages (Fig. 5C). As expected, *AQP1* expression was not detected by qRT-PCR in any of the three other mutants (Fig. 5C).

**Fig 6:**
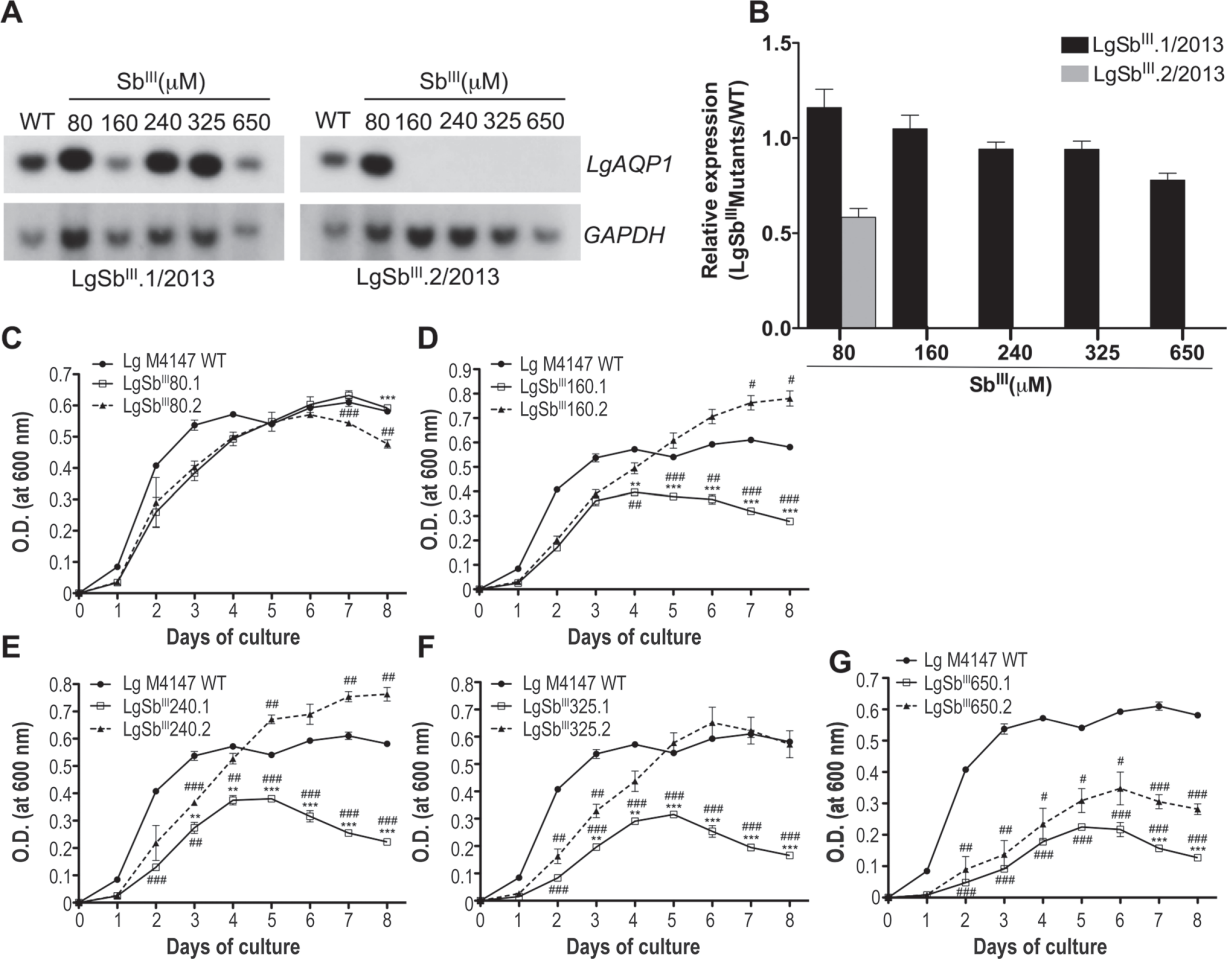
Kinetics of *LgAQP1* loss. (A) Southern blots of *Pst*I-digested genomic DNA derived from the LgSb^III^.1/2013 (left) and LgSb^III^.2/2013 (right) series of *L*. *guyanensis* SbR mutants hybridized with a *LgAQP1* probe. G*APDH* signals were used as DNA loading control. (B) Relative mRNA levels of LgAQP1 in LgSb^III^/2013 mutants compared to LgM4147 WT. SbR/WT expression ratios were normalized according to *GAPDH* levels. Values are the mean of two independent experiments each performed in three biological replicates. The growth of intermediate step LgSb^III^/2013 mutants selected at 80 μM (C), 160 μM (D), 240 μM (E), 325 μM (F) and 650 μM (G) was monitored in the presence of appropriate Sb^III^ concentrations. LgM4147 WT growth was monitored in the absence of Sb^III^. An asterisk (*) indicates comparison between LgSb^III^.1/2013 and LgSb^III^.2/2013, while the # symbol refers to comparison between a SbR mutant and the WT parent. Values represent the average of two independent growth measurements performed in duplicate. Statistical analysis was carried out using Student’s t-test. * or # *p* ≤ 0.05, ** or ## *p* ≤ 0.01 and *** or ### *p* ≤ 0.001.

To better understand the kinetics of *AQP1* deletion and its implication on growth fitness in the presence and absence of drug pressure, we selected two new series of SbR *L*. *guyanensis* mutants by five Sb^III^ increments until reaching a final concentration of 650 μM. These series were named LgSb^III^.1/2013 and LgSb^III^.2/2013 (Table 1). At the first selection step (80 μM) *AQP1* remained unaltered in both cell lines (Fig. 6A). When Sb^III^ selection was increased, the *AQP1* gene remained intact in the LgSb^III^.1/2013 series but was lost in mutant LgSb^III^160.2/2013 already at 160 μM (Fig. 6A). The amount of *AQP1* mRNA was consistent with the copy number of the gene (Fig. 6B). Growth curves of LgSb^III^.1/2013 and LgSb^III^.2/2013 mutants revealed an advantage associated with the loss of *AQP1* when parasites were cultivated in the presence of Sb^III^. Indeed, the LgSb^III^.2/2013 mutant without *AQP1* grew faster under Sb^III^ selection (up to 325 μM) than LgSb^III^.1/2013 mutants (Fig. 6D to 6F) presenting intact *AQP1* copies (Figs. 6A and S3). This growth advantage of LgSb^III^.2/2013 was not observed when parasites were cultures in drug free medium (S4 Fig.).

### Single mutation G133D in AQP1 is involved in antimony resistance in *L*. *guyanensis*

**Fig 7:**
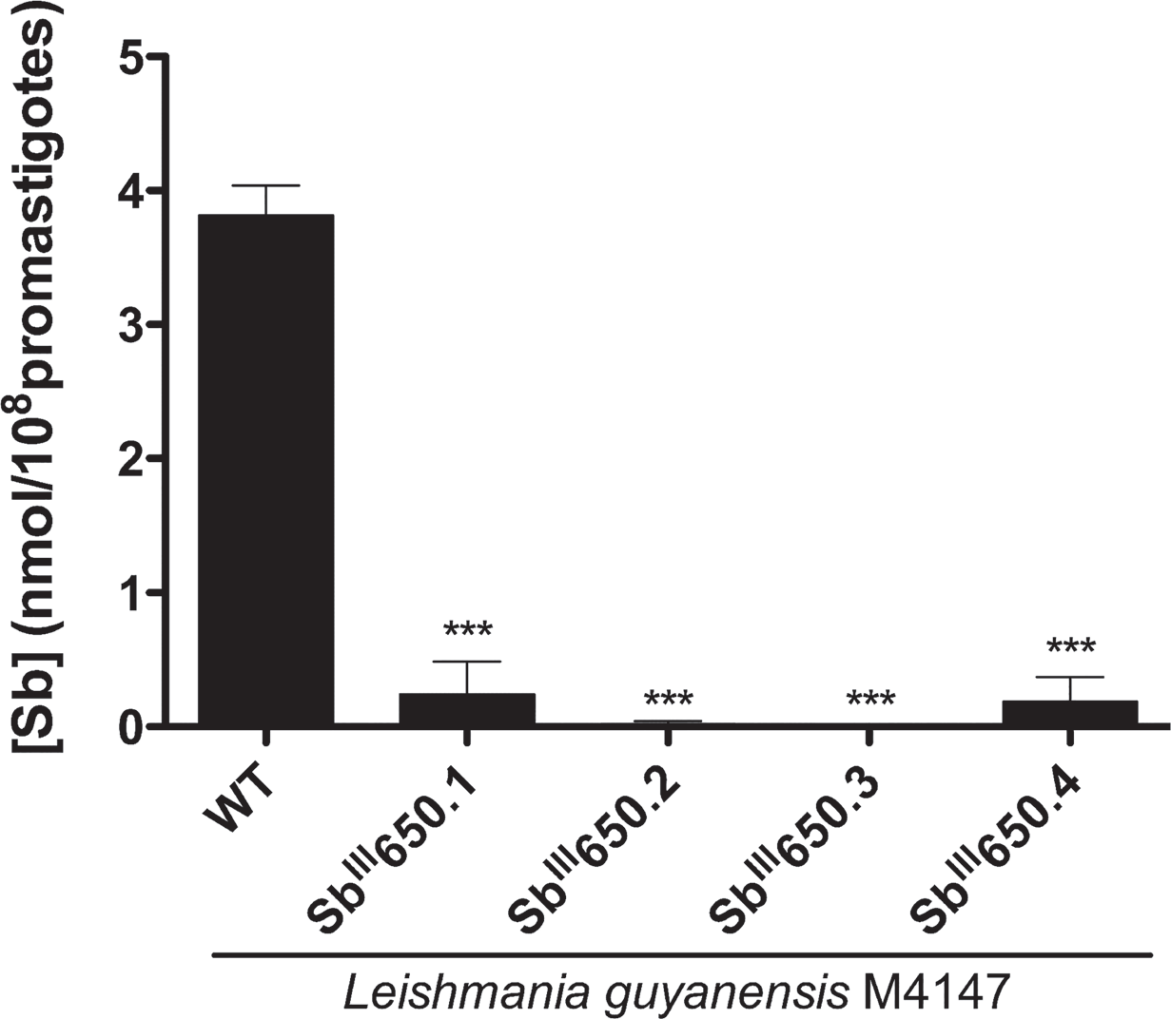
Intracellular antimony accumulation in *L*. *guyanensis* parasites sensitive and resistant to antimony. Antimony quantification was performed using atomic absorption after 1 h incubation of LgWT and LgSb^III^650.1–4 parasites with 540 μM of Sb^III^. Values were obtained from two independent experiments performed in quadruplicate and represent the mean antimony concentration ± SEM. Statistical analysis were carried out using ANOVA followed by Bonferroni’s multiple comparison test. *** *p* ≤ 0.0001.

**Fig 8:**
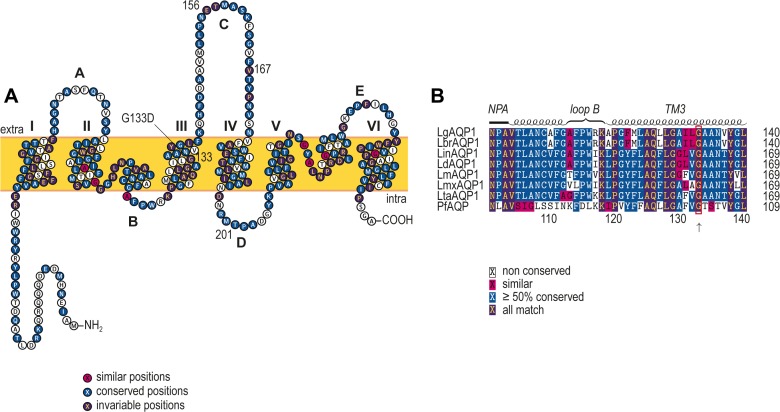
Putative LgAQP1 topology and sequence comparisons of *Leishmania* AQP1 orthologues. (A) Topology prediction for LgAQP1 based on previously published predicted structures of LmAQP1 [71] and PfAQP [77,78]. Color-code consensus is based on the alignment presented in panel B. The single mutation G133D is indicated in transmembrane domain III and is conserved in all organisms. (B) Multiple sequence alignment of a selected AQP1 region from seven *Leishmania* species and from *Plasmodium falciparum* AQP. Topology and alignment were respectively plotted using TEXtopo and TEXshade LaTeX2e macro packages [79,80]. Lg, *L*. (*V*.) guyanensis; Lbr, *L*. (*V*.) *braziliensis*; Lin, *L*. (*L*.) *infantum*; Ld, *L*. (*L*.) *donovani*; Lm, *L*. (*L*.) *major*; Lmx, *L*. (*L*.) *mexicana*; Lta, *L*. (*S*.) *tarentolae*; Pf, *Plasmodium falciparum*.

While the loss of *AQP1* allows for a faster acquisition of resistance (Fig. 6), mutant LgSb^III^650.4 had intact *AQP1* copy number (Fig. 5B) and expression levels (Fig. 5C). Antimony accumulation experiments were thus performed with the *L*. *guyanensis* mutants. Quantification of intracellular antimony in *L*. *guyanensis* revealed, as expected, a very low accumulation of metalloid in the *L*. *guyanensis* SbR mutants in which *AQP1* was deleted when compared to WT parasites (Fig. 7). Surprisingly, we also observed low accumulation in LgSb^III^650.4 (Fig. 7). We hypothesized that *AQP1* in LgSb^III^650.4 may be mutated and this was confirmed by sequencing the gene, which revealed a single nucleotide polymorphism (SNP) at *AQP1* position 398 in LgSb^III^650.4, substituting a guanine for an adenine (S5 Fig.). This missense mutation in *AQP1* translated into the replacement of a glycine (Gly) residue by an aspartic acid (Asp) at position 133 (G133D) of the protein in LgSb^III^650.4 (S6 Fig.). Glycine 133 is putatively located in the third transmembrane domain in LgAQP1 (Fig. 8A) and is conserved among several *Leishmania* species and also in the *Plasmodium falciparum* AQP (PfAQP) (Fig. 8B).

**Fig 9:**
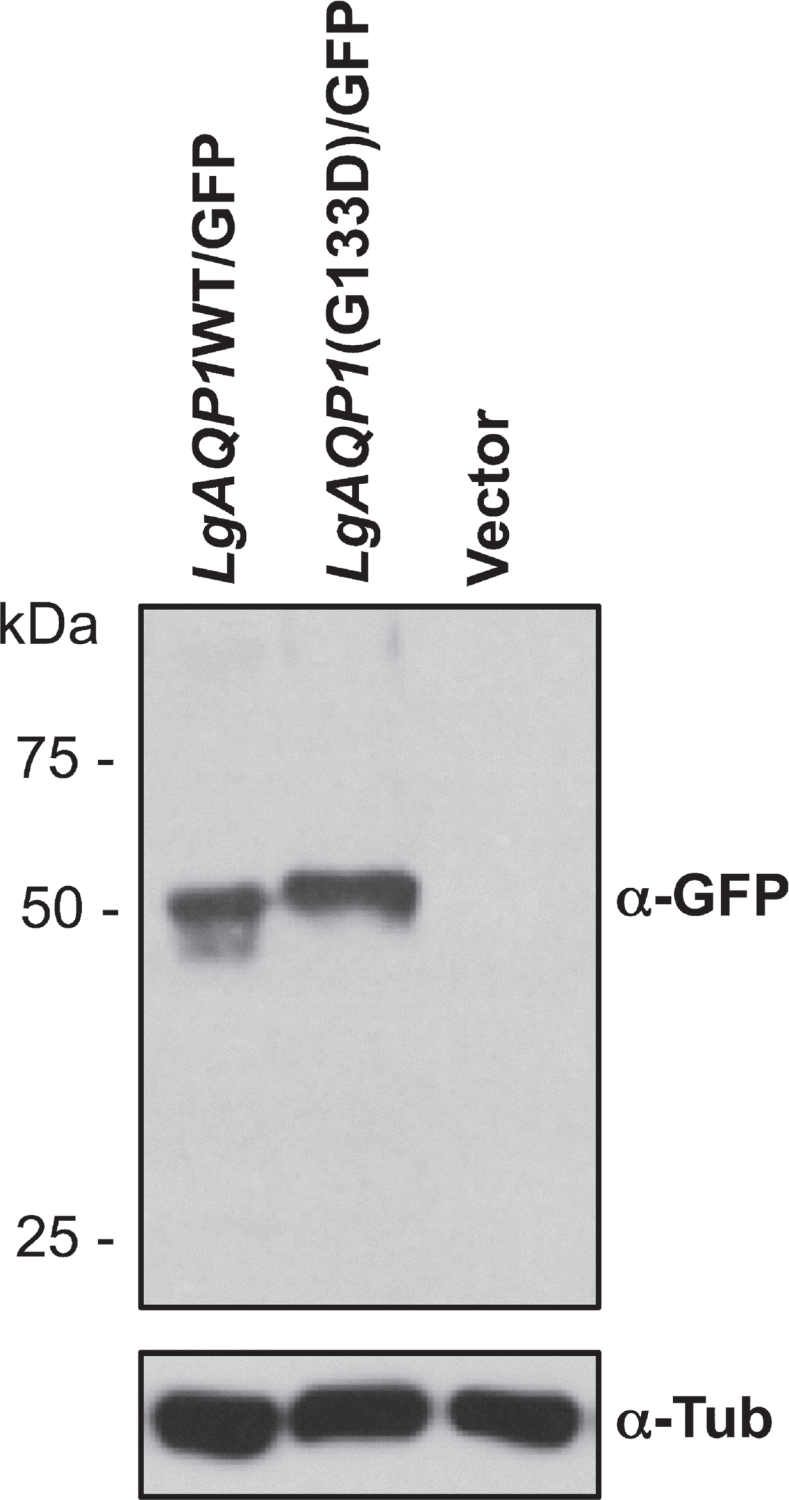
Analysis of LgAQP1-GFP expression by Western blot. Anti-GFP monoclonal antibody was used to confirm the expression of the LgAQP1WT-GFP and LgAQP1G133D-GFP fusion proteins from protein extracts prepared from LgSb^III^650.2 parasites transfected with pSP72αZEOαLgAQP1WT (lane 1), pSP72αZEOαLgAQP1G133D (lane 2) or pSP72αZEOα (lane 3). Anti-α-tubulin antiserum was used as a loading control.

To functionally validate the contribution of the AQP1 G133D mutation in antimony resistance in *L*. *guyanensis*, GFP-tagged version of AQP1WT and AQP1G133D were episomally maintained in LgSb^III^650.2, which is naturally disrupted for *AQP1* (Fig. 5B). Hybridization of Western blots with an antibody directed against GFP yielded the expected 50 kDa band for the fusion protein and confirmed the overexpression of the fusion protein in the respective LgSb^III^650.2 transfectants (Fig. 9). The overexpression of LgAQP1WT substantially sensitized LgSb^III^650.2 to Sb^III^ whose EC_50_ dropped from more than 1000 μM in the mock-transfected control, to 29 μM in the presence of the WT AQP1 allele (Table 1). On the other hand, LgSb^III^650.2 transfected with an AQP1 version harboring the G133D mutation remained as resistant as the mock-transfected control (Table 1). The presence of GFP did not interfere with the function of AQP1, as the tagged version of WT AQP1 was equally potent as an untagged version of the protein at sensitizing the LgSb^III^650.2 mutant to Sb^III^ (Table 1). The WT version of *AQP1* but not its mutated version also restored Sb^III^ sensitivity when transfected in LgSb^III^650.2 (Table 1). The G133D AQP1 also failed to alter Sb^III^ EC_50_ when overexpressed in a *L*. *guyanensis* WT background (Table 1).

## Discussion

Next generation sequencing has been a useful approach for studying drug resistance in *Leishmania* parasites for detecting both point mutations and changes in copy number of genes [30,31,52,56–58]. A frequent mechanism of drug resistance is gene amplification of specific regions that happens at the levels of repeated sequences that abound in the *Leishmania* genomes [55]. Changes in copy number can extend also to whole chromosomes [30,33,59] and it has been argued that tolerance of such chromosomal CNVs may be beneficial under stress conditions as in the presence of drug pressure [60]. Finally the individual parasites within a population may have different specific genes amplified [55] and may have different ploidy of specific chromosomes [53,61]. The NGS technology was useful to detect several ploidy changes in *Leishmania* species and here we have tested it with the *Viannia* subgenus. Normalized read depth coverage identified chromosomes in our SbR *L*. *guyanensis* mutants whose ploidy was altered compared to WT parasites (Fig. 1). Recurrent changes are often strong candidates for linking a phenotype to a genotype and it is salient to point out that no single chromosome ploidy was identical between the 4 mutants (Fig. 1). The link between aneuploidy and drug resistance might therefore be circumstantial, but antimony resistance is a complex and multifactorial process [29] and, in this context, studying the cellular consequences of aneuploidy might still provide novel insights on drug resistance in *Leishmania*.

Sequence reads corresponding to a subtelomeric region of chromosome 19 were higher in three mutants out of four (Fig. 2A). This was confirmed by Southern blots, but chromosome sized gels did not support the possibility that this region was amplified as part of an extrachromosomal element and instead consisted in an intrachromosomal duplication (Fig. 2C and 2D). While representing a rare event in *Leishmania* compared to extrachromosomal amplification, this has already been observed while attempting to inactivate the essential gene GSH1 in *Leishmania* [62] or in *L*. *major* cells resistant to antimony in which an intrachromosomal amplification of a subtelomeric region of chromosome 34 was observed [31]. Species belonging to the *Viannia* subgenus have previously been reported to display a limited capacity to generate/maintain extrachromosomal DNA [63,64] which is consistent with the intrachromosomal amplifications observed here. Nonetheless, episome transfection is possible in *Viannia* (see Table 1) [65] and in some studies gene amplification was observed in *Viannia* parasites [38,66].

Read depth coverage also revealed large regions of chromosome 23 encompassing the *MRPA* resistance locus that were amplified in the four resistant mutants (Fig. 3A), a feature confirmed by hybridization of Southern blots (Fig. 3B). Karyotype analyses by PFGE revealed a new band of ~ 1 Mb in three mutants and an apparent change in chromosome ploidy for the fourth mutant (Fig. 3C and 3D). The exact mechanism of formation of the 1 Mb band is unknown but a fragment of 495 kb derived from chromosome 23 must have been rearranged since the MRPA and *LbrM*.*23*.*1000* probes are 495 kb apart (Fig. 3C and 3D). The hybridization signal to the novel ~ 1 Mb band in LgSb^III^650.1, LgSb^III^650.2 and LgSb^III^650.3 is less intense then the native chromosome 23 signal at ~ 800 kb, suggesting that the duplication of the MRPA containing fragment may have happened in only part of the population. Because of the short reads linked to Illumina sequencing, it is not helpful in determining how a portion of chromosome 23 has duplicated into a larger chromosome. One possibility would involve translocation (possibility subtelomeric) from one chromosome to another. While gene amplification (extrachromosomal or intrachromosomal) usually occurs at the level of repeated sequences [55], we have reported rare mechanistic events leading to gene amplification [31] and further studies are required to explain how these *MRPA* amplifications are produced. These results are consistent with the data shown above for the subtelomeric region of chromosome 19 where in *Viannia*, in comparison to *Leishmania*, increase in copy number is mediated by mechanisms that do not involve extrachromosomal amplification.

A terminal deletion of ~ 20 kb of seven genes on chromosome 31 including the gene coding for AQP1 was observed in three mutants (Fig. 5A and B). AQP1 is considered the major route of entry of trivalent antimony in *Leishmania* [27] and its overexpression leads to Sb^III^ hypersensitivity [26,27]. Downregulation of *AQP1* has been observed in both laboratory-raised and clinical *Leishmania* parasites resistant to antimony [26,31,67,68] and constitutes a potentially useful biomarker for antimony resistance. Deletion of *AQP1* is also a major contributor to SbR in *L*. *guyanensis* because episomal overexpression of a WT *AQP1* allele was sufficient to restore Sb sensitivity (Table 1) and accumulation (Fig. 7) in every mutant tested. The deletion of the gene also suggests that AQP1 is not essential in *L*. *guyanensis*. Chromosome 31 is polyploid in all *Leishmania* spp. tested [30,52,60] and the 20 kb region was deleted from all chromosome copies (Fig. 5B). Terminal deletions from 67 to 205 kb covering the *AQP1* locus on chromosome 31 were also recently observed in SbR *L*. *major* for which the break points occurred at the level of inverted repeated sequences [31]. In these cases however, there was still one intact copy of *AQP1*. In contrast, *L*. *guyanensis* SbR mutants presented deleted regions of only 20.7 to 22.7 kb on chromosome 31 without any sign of inverted repeated sequences. The terminal deletion could thus be driven by micro-homologies with telomere-associated repeated sequences [31,69] or through a double strand break followed by a terminal healing process driven by telomere seeding [70], although any of these mechanisms need to be ascertain.

In one mutant, the *AQP1* gene was not deleted but transport experiments indicated that there was no accumulation of Sb^III^ (Fig. 7). This prompted us to sequence *AQP1* and to demonstrate for the first time that a point mutation in AQP1 (G133D) can also be a novel resistance mechanism. Mutational analysis on LmAQP1 had already revealed that residues located at C-loop Ala163 and Glu152 (equivalent to LgAQP1 residues Val167 and Glu156, respectively—Fig. 8A) are involved in metalloid uptake and reduced permeability to antimony [28,71]. LmAQP1 is also post-transcriptionally regulated by a mitogen activated protein kinase 2-mediated phosphorylation at Thr197 (LgAQP1 Thr201—Fig. 8A) modulating Sb^III^ uptake and sensitivity [72]. The absence of a crystal structure for *Leishmania* AQP1 precludes hypothesizing about the precise role of G133D in resistance. The lack of antibodies and the low level of GFP fluorescence have not allowed to test whether the G133D mutation could also impact the subcellular localization of AQP1. Interestingly, the homolog of LgAQP1 in the related protozoan *Trypanosoma brucei* (TbAQP2) was also linked to resistance to the arsenical-based compound melarsoprol (arsenic is a metalloid chemically related to antimony) [73,74]. Melarsoprol-resistant *T*. *brucei* were shown to have lost *TbAQP2* or to harbor a nonfunctional chimera derived from recombination events between *TbAQP2* and *TbAQP3* [75] which show similarity with the mutants studied here (deletion or point mutations). Given the important role of aquaglyceroporins in volume and osmotaxis regulation [25], how null mutants compensate these functions is still an open question and further studies will be required for understanding their physiological adaptations.

Every *L*. *guyanensis* SbR mutants had a defect in antimony accumulation. Lower accumulation can be achieved either through decreased uptake or increased efflux. An additional transport mechanisms leading to resistance would be drug sequestration mediated by the intracellular ABC protein MRPA [36]. The lack of a functional AQP1 will lead to reduced uptake. Possibly minimal amounts of antimony can enter by other routes and overexpression of MRPA can lead to drug sequestration. Alternatively, the contribution of MRPA to resistance may be more important in early selection steps when all *AQP1* copies (chromosome 31 harbouring *AQP1* is polyploidy in every *Leishmania* species) have not yet been inactivated/deleted. It is salient to point out that alterations in expression of MRPA and AQP1 has been described in antimony-resistant natural isolates of *L*. *donovani* [29,67,68] and *L*. *tropica* [76], suggesting a concomitant antimony sequestering and decreased uptake [67,76]. These results are also consistent with antimony resistant *L*. *amazonensis* mutants selected *in vitro* [38]. The loss of AQP1 appears to be dominant in our current mutants since providing the mutants with a functional version of *AQP1* enables complete re-sensitization to SbIII.

The present study highlighted that similar markers are involved in resistance in *Leishmania* and *Viannia* subgenus but that gene amplification differs with mostly extrachromosoal amplicons in *Leishmania* and intrachromosomal ones in *Viannia*. A new resistance mechanism corresponding to a point mutation in AQP1 was also discovered and this will allow further testing of the role of AQP1 in resistance.

## Supporting Information

S1 Fig Normalized SbR/WT reads ratios per non-overlapping 5kb genomic window for the 35 chromosomes of *L*. *guyanensis*. Chromosomes were divided into non-overlapping 5kb genomic windows and for each window the SbR/WT reads ratios (normalized to the total number of reads per samples) were plotted as log2-transfomed values according to chromosome positions. Blue, LgSb^III^650.1; Red, LgSb^III^650.2; Green, LgSb^III^650.3; and Yellow, LgSb^III^650.4.
(ZIP)

S2 Fig Scheme representation of the *MRPA* locus on chromosome 23. Grey and black arrows indicate pairs of inverted repeats that could anneal to prime the inverted duplication of the large 495 kb region. Black arrows annealing would result in a 1,032 kb linear element while grey arrows would lead to a 1,123 kb linear amplicon.
(TIF)

S3 Fig Multiple nucleotide alignment of *AQP1* from the LgSb^III^.1/2013 series of mutants. *AQP1* sequences were compared to *L*. *guyanensis AQP1* GenBank accession number GU368155.1 (LgAQP1_GB). Alignment was performed using the MultiAlin interface (multialin.toulouse.inra.fr).
(TIF)

S4 Fig Growth curves of LgSb^III^/2013 mutants and Lg M4147 WT in absence of Sb^III^. The growth of LgSb^III^.1/2013 and LgSb^III^.2/2013 mutants resistant to 80 μM (A), 160 μM (B), 240 μM (C), 325 μM (D) and 650 μM (E) Sb^III^ was monitored without drug pressure for 8 days. The growth of the parental LgM4147 WT line was also monitored. An asterisk (*) indicates comparisons between LgSb^III^.1/2013 and LgSb^III^.2/2013 mutants. Values represent the average of two independent growth measurements performed in duplicate. Statistical analysis was carried out using Student’s t-test. * *p* ≤ 0.05 and ** *p* ≤ 0.01.
(TIF)

S5 Fig Multiple nucleotide alignment of *AQP1* from *L*. *guyanensis* M4147 WT and LgSb^III^650.4. Guanine 398 is replaced by an adenine in LgSb^III^650.4. The *L*. *guyanensis AQP1* sequence GU368155.1 (strain MHOM/BR/1997/NMT-MAO 328P clone B) was used as an additional reference [81]. Alignment was performed with ClustalW2 and plotted using TEXshade [79,80]. *L*. *guyanensis AQP1* sequences are available in GenBank, accession numbers KJ623262 and KJ623263.
(TIF)

S6 Fig Multiple alignment of AQP1 protein sequences from *L*. *guyanensis* M4147 WT and LgSb^III^650.4. The substitution of a glycine by an aspartic acid at position 133 (G133D) is associated with antimony resistance in mutant LgSb^III^650.4. The *L*. *guyanensis* AQP1 sequence GU368155.1 was used as an additional reference [81]. Alignment was performed using ClustalW2 and plotted using TEXshade [79,80].
(TIF)

S1 Table List of PCR primers used in this study. (TIF)
