# Supplementary figures and images for: Intrachromosomal Amplification, Locus Deletion and Point Mutation in the Aquaglyceroporin AQP1 Gene in Antimony Resistant Leishmania (Viannia) guyanensis
Source: PLoS Negl Trop Dis. 2015 Feb 13;9(2):e0003476. doi: 10.1371/journal.pntd.0003476 (PMC4332685; doi:10.1371/journal.pntd.0003476)

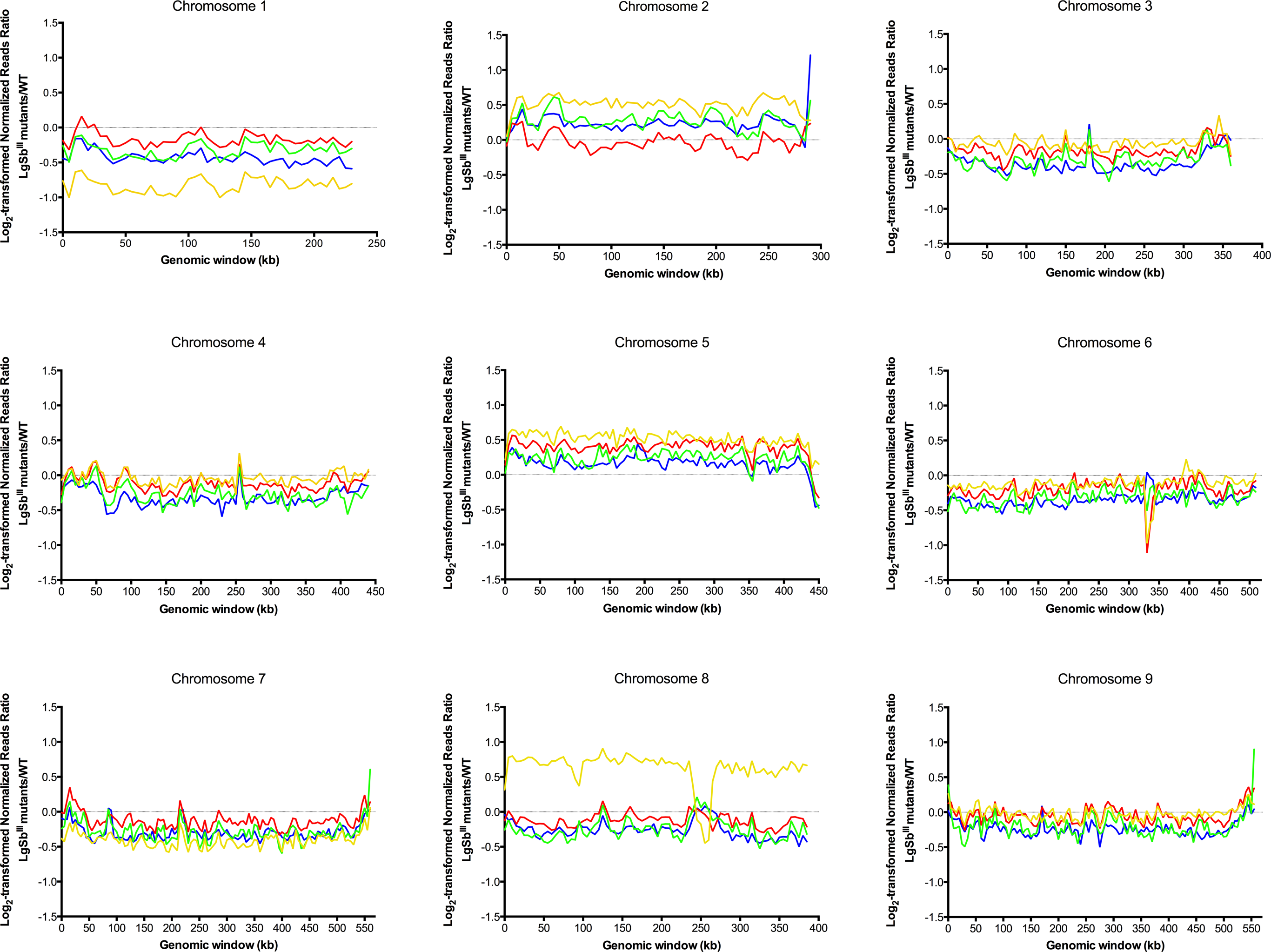

Supplement: S1 Fig — Chromosomes were divided into non-overlapping 5kb genomic windows and for each window the SbR/WT reads ratios (normalized to the total number of reads per samples) were plotted as log2-transfomed values according to chromosome positions. Blue, LgSbIII650.1; Red, LgSbIII650.2; Green, LgSbIII650.3; and Yellow, LgSbIII650.4. (ZIP) [file pntd.0003476.s001.zip › Figure S1/Figure S1a.tif]

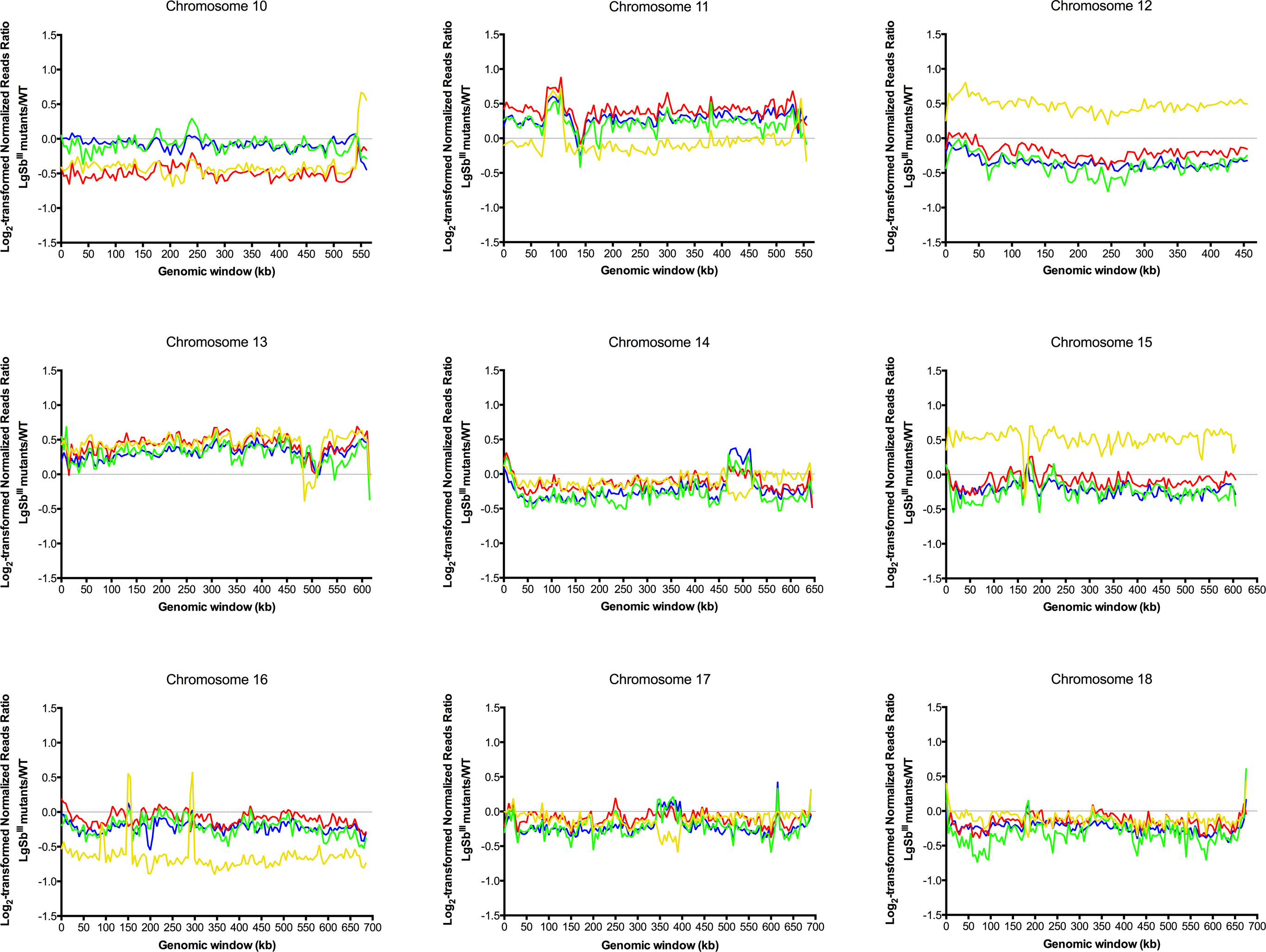

Supplement: S1 Fig — Chromosomes were divided into non-overlapping 5kb genomic windows and for each window the SbR/WT reads ratios (normalized to the total number of reads per samples) were plotted as log2-transfomed values according to chromosome positions. Blue, LgSbIII650.1; Red, LgSbIII650.2; Green, LgSbIII650.3; and Yellow, LgSbIII650.4. (ZIP) [file pntd.0003476.s001.zip › Figure S1/Figure S1b.tif]

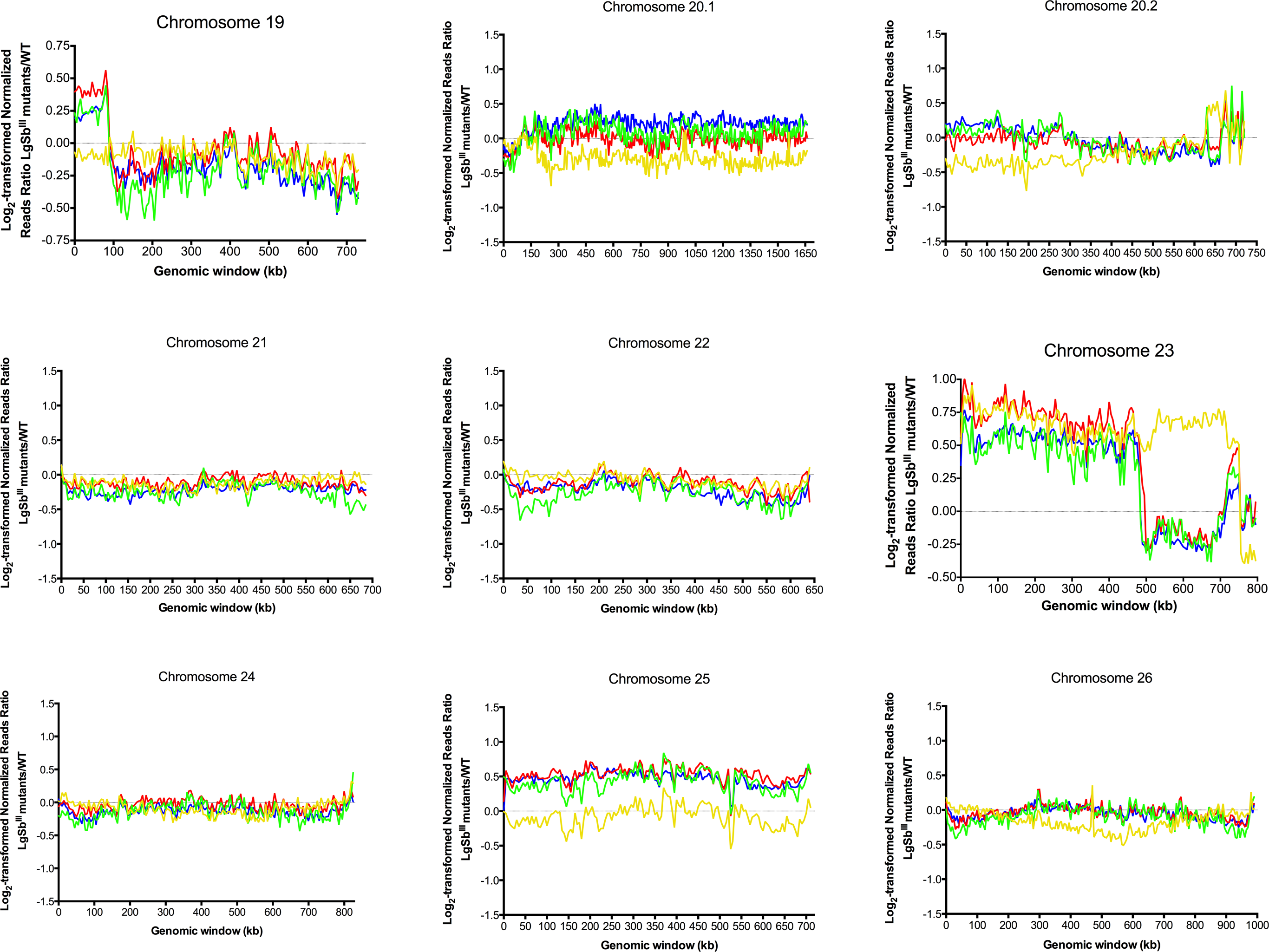

Supplement: S1 Fig — Chromosomes were divided into non-overlapping 5kb genomic windows and for each window the SbR/WT reads ratios (normalized to the total number of reads per samples) were plotted as log2-transfomed values according to chromosome positions. Blue, LgSbIII650.1; Red, LgSbIII650.2; Green, LgSbIII650.3; and Yellow, LgSbIII650.4. (ZIP) [file pntd.0003476.s001.zip › Figure S1/Figure S1c.tif]

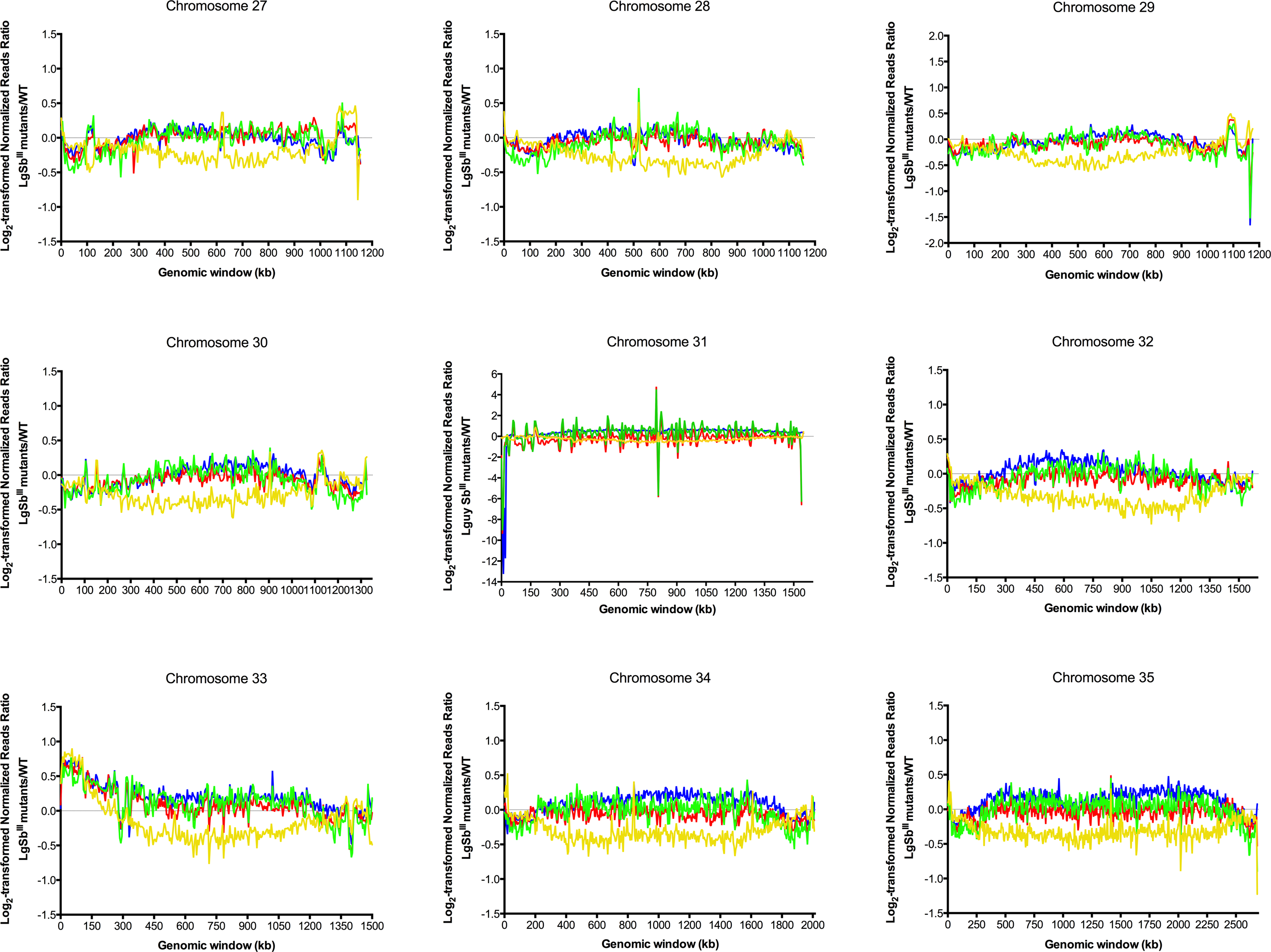

Supplement: S1 Fig — Chromosomes were divided into non-overlapping 5kb genomic windows and for each window the SbR/WT reads ratios (normalized to the total number of reads per samples) were plotted as log2-transfomed values according to chromosome positions. Blue, LgSbIII650.1; Red, LgSbIII650.2; Green, LgSbIII650.3; and Yellow, LgSbIII650.4. (ZIP) [file pntd.0003476.s001.zip › Figure S1/Figure S1d.tif]

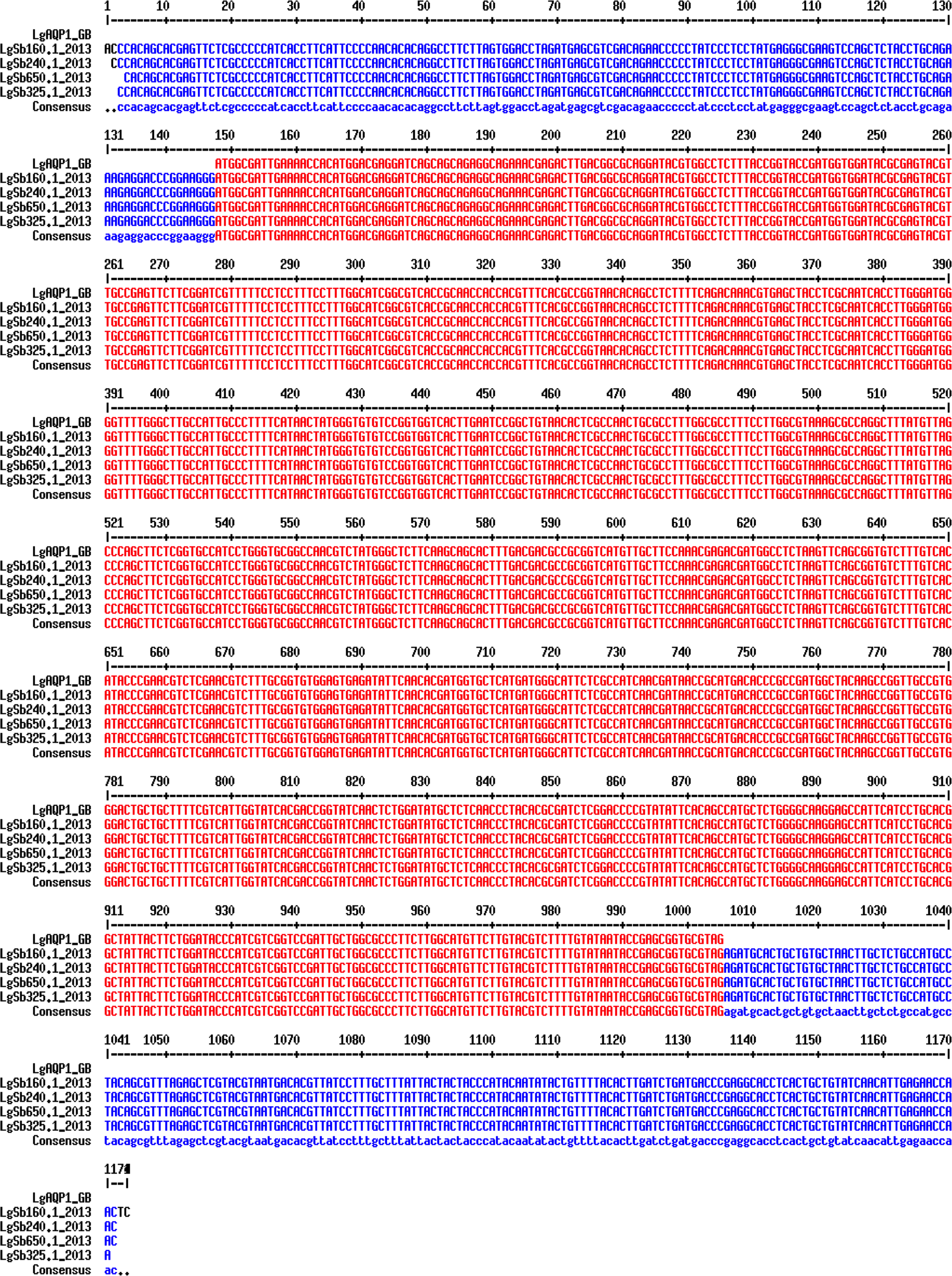

Supplement: S3 Fig — AQP1 sequences were compared to L. guyanensis AQP1 GenBank accession number GU368155.1 (LgAQP1_GB). Alignment was performed using the MultiAlin interface (multialin.toulouse.inra.fr). (TIF) [file pntd.0003476.s003.tif]

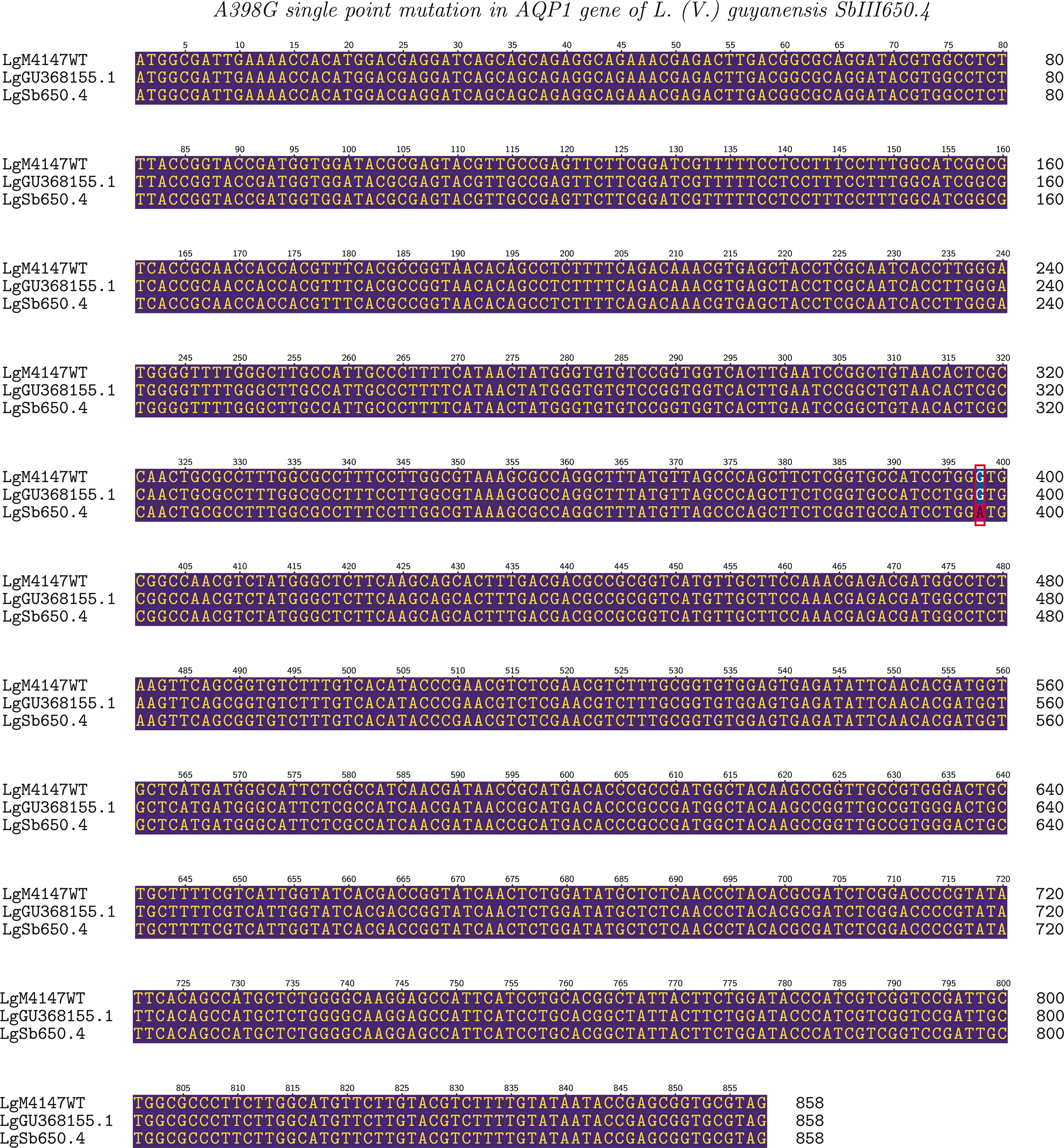

Supplement: S5 Fig — Guanine 398 is replaced by an adenine in LgSbIII650.4. The L. guyanensis AQP1 sequence GU368155.1 (strain MHOM/BR/1997/NMT-MAO 328P clone B) was used as an additional reference [81]. Alignment was performed with ClustalW2 and plotted using TEXshade [79,80]. L. guyanensis AQP1 sequences are available in GenBank, accession numbers KJ623262 and KJ623263. (TIF) [file pntd.0003476.s005.tif]

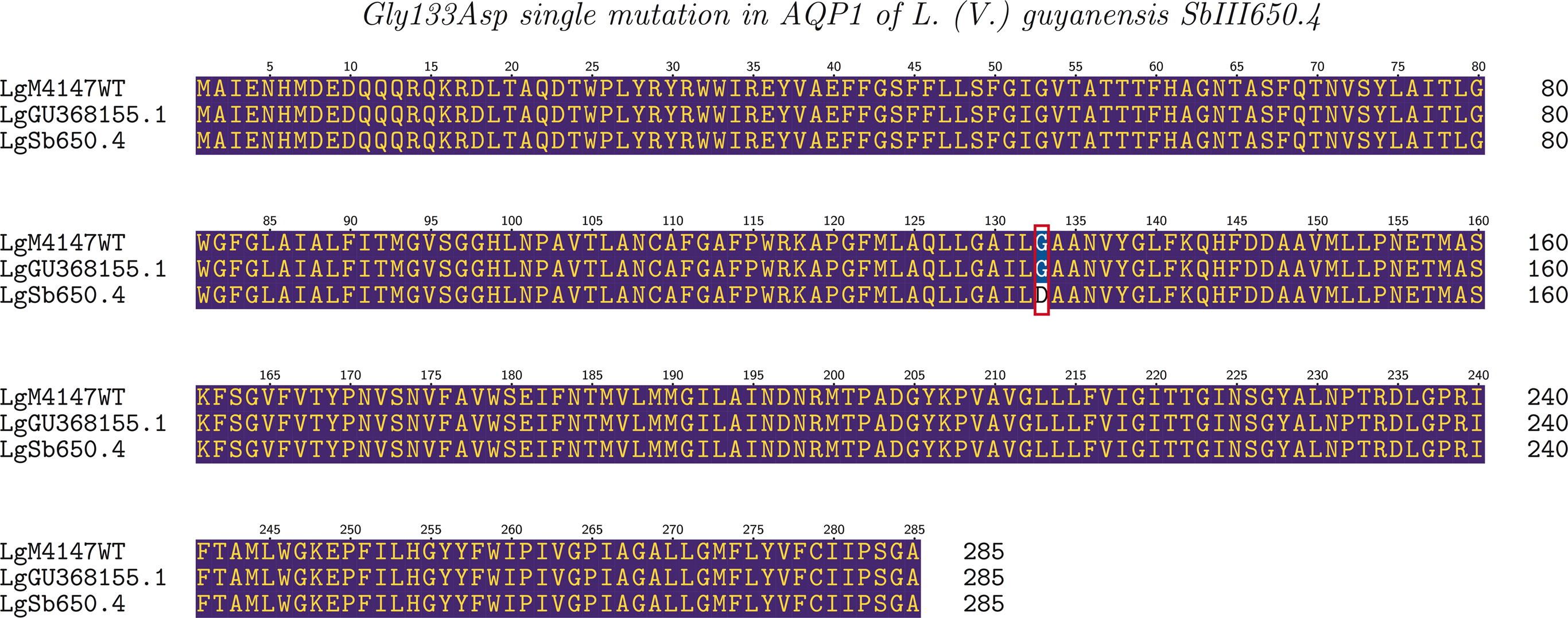

Supplement: S6 Fig — The substitution of a glycine by an aspartic acid at position 133 (G133D) is associated with antimony resistance in mutant LgSbIII650.4. The L. guyanensis AQP1 sequence GU368155.1 was used as an additional reference [81]. Alignment was performed using ClustalW2 and plotted using TEXshade [79,80]. (TIF) [file pntd.0003476.s006.tif]
